# Digital Health Interventions to Enhance Prevention in Primary Care: Scoping Review

**DOI:** 10.2196/33518

**Published:** 2022-01-21

**Authors:** Van C Willis, Kelly Jean Thomas Craig, Yalda Jabbarpour, Elisabeth L Scheufele, Yull E Arriaga, Monica Ajinkya, Kyu B Rhee, Andrew Bazemore

**Affiliations:** 1 Center for Artificial Intelligence, Research, and Evaluation IBM Watson Health Cambridge, MA United States; 2 Policy Studies in Family Medicine and Primary Care The Robert Graham Center American Academy of Family Physicians Washington, DC United States; 3 The American Board of Family Medicine Lexington, KY United States

**Keywords:** digital technology, primary health care, preventive medicine, telemedicine, clinical decision support systems

## Abstract

**Background:**

Disease prevention is a central aspect of primary care practice and is comprised of primary (eg, vaccinations), secondary (eg, screenings), tertiary (eg, chronic condition monitoring), and quaternary (eg, prevention of overmedicalization) levels. Despite rapid digital transformation of primary care practices, digital health interventions (DHIs) in preventive care have yet to be systematically evaluated.

**Objective:**

This review aimed to identify and describe the scope and use of current DHIs for preventive care in primary care settings.

**Methods:**

A scoping review to identify literature published from 2014 to 2020 was conducted across multiple databases using keywords and Medical Subject Headings terms covering primary care professionals, prevention and care management, and digital health. A subgroup analysis identified relevant studies conducted in US primary care settings, excluding DHIs that use the electronic health record (EHR) as a retrospective data capture tool. Technology descriptions, outcomes (eg, health care performance and implementation science), and study quality as per Oxford levels of evidence were abstracted.

**Results:**

The search yielded 5274 citations, of which 1060 full-text articles were identified. Following a subgroup analysis, 241 articles met the inclusion criteria. Studies primarily examined DHIs among health information technologies, including EHRs (166/241, 68.9%), clinical decision support (88/241, 36.5%), telehealth (88/241, 36.5%), and multiple technologies (154/241, 63.9%). DHIs were predominantly used for tertiary prevention (131/241, 54.4%). Of the core primary care functions, comprehensiveness was addressed most frequently (213/241, 88.4%). DHI users were providers (205/241, 85.1%), patients (111/241, 46.1%), or multiple types (89/241, 36.9%). Reported outcomes were primarily clinical (179/241, 70.1%), and statistically significant improvements were common (192/241, 79.7%). Results were summarized across the following 5 topics for the most novel/distinct DHIs: population-centered, patient-centered, care access expansion, panel-centered (dashboarding), and application-driven DHIs. The quality of the included studies was moderate to low.

**Conclusions:**

Preventive DHIs in primary care settings demonstrated meaningful improvements in both clinical and nonclinical outcomes, and across user types; however, adoption and implementation in the US were limited primarily to EHR platforms, and users were mainly clinicians receiving alerts regarding care management for their patients. Evaluations of negative results, effects on health disparities, and many other gaps remain to be explored.

## Introduction

The Institute of Medicine declared primary care to be “essential health care” and the central feature of an effective health care system [1]. Primary care has the potential to enhance quality, reduce costs, and increase equity and access to care by providing first contact and easy access to comprehensive, continuous, and coordinated medical care for patients [2] and populations, as articulated in the 4Cs framework by Dr Barbara Starfield [3]. Prevention of diseases and their complications ranks among primary care’s most fundamental functions; when performed effectively, primary care prevention can decrease mortality and morbidity in both chronic and acute conditions [4]. Various practitioners, including physicians, nurses, physician assistants, and pharmacists, recognize its value, but preventive services are often underutilized [5], despite guideline recommendations provided by the US Preventive Services Task Force [6].

Many studies have investigated the sources of suboptimal preventive health service delivery. Among the major barriers to preventive care implementation by clinicians is time. Studies have shown that 8.6 hours per working day are needed for a clinician to fully satisfy the US Preventive Services Taskforce preventive care recommendations for their patients [7]. A steady growth in competing demands across the management of acute, chronic, and preventive needs and an aging population with increasing comorbidities make it nearly impossible for a clinician to provide recommended preventive services without support. Innovations in care delivery, such as the patient-centered medical home [8], use of community health workers [9], and integration of primary care with public health [10], can help reduce this burden on clinicians, but with the rapid evolution of information technology, digital health interventions (DHIs) to address prevention are crucial.

DHIs are delivered via digital technologies to support a variety of health system needs and are used both formally and informally by providers, patients, and population stakeholders. Examples of these technologies include mobile wireless health devices (mobile health [mHealth]) using SMS or smartphone apps, telehealth systems for remote clinical services, wireless medical devices, software as a medical device (eg, clinical decision support), medical imaging, health information technology (HIT), and patient portals. Other digital health facets, such as advanced data analytics and artificial intelligence (AI), may be used as standalone interventions or integrated components within digital technologies. Digital health technologies may or may not be regulated by the US Food and Drug Administration (FDA) or recognized by the World Health Organization (WHO).

DHIs can support primary (eg, timely receipt of vaccinations), secondary (eg, completion of indicated screenings), tertiary (eg, routine monitoring of chronic conditions), and quaternary (eg, prevention of overmedicalization) prevention. DHIs have provided meaningful outcomes via the incorporation of care management programs, disease registries, and behavioral change interventions to improve medication adherence, promote weight loss, support smoking and substance abuse cessation, and enhance mental health [11]. Moreover, DHIs have been effectively used to address racial, ethnic, and socioeconomic health disparities [12]. In addition, the COVID-19 pandemic has accelerated the adoption of DHIs, such as telehealth services, and raised the possibility of longer-term incorporation of such technologies by a primary care community that has traditionally lagged hospital and acute care peers.

Although prior studies have examined the impact of individual DHIs on preventive service receipt, no comprehensive review of these modalities exists to date. A scoping review with a subgroup analysis was conducted to understand how DHIs are being used in US primary care settings to enhance and support the delivery of preventive care.

## Methods

### Study Design

A scoping review was conducted in accordance with the PRISMA-ScR (Preferred Reporting Items for Systematic Reviews and Meta-Analyses extension for Scoping Reviews) guidelines [13] to identify studies that examined patients/consumers, providers, and/or population stakeholders in primary care settings (eg, limited to outpatient, ambulatory care, and long-term care) that used at least one digital health technology as an intervention for prevention (primary [eg, timely receipt of vaccinations], secondary [eg, completion of indicated screenings], tertiary [eg, routine monitoring of chronic conditions], and quaternary [eg, prevention of overmedicalization]) and reported beneficial outcomes on health, health care performance, and implementation science. The protocol is available upon request.

### Search Strategy

Systematic search queries of MEDLINE via PubMed, Embase, and the Cochrane Library were used to identify references published or available online between January 1, 2014, and July 19, 2020 (Multimedia Appendices 1-7). Studies were limited to primary designs or systematic reviews (with the same inclusion criteria) published in English with abstracts. The rationale for this search cutoff time frame was based upon a high threshold of eligible providers achieving meaningful use of certified electronic health record (EHR) technology, whereby 82.8% of office-based physicians had adopted any EHR [14].

### Screening Process

To ensure screener alignment, dual review of 20% of randomized titles and abstracts was followed by group resolution of conflicts. All remaining titles and abstracts underwent single review, and full-text articles were examined by 2 independent reviewers for relevance against the inclusion/exclusion criteria (Multimedia Appendix 8), with third-party adjudication provided for any discrepancies in eligibility. Results were tracked in DistillerSR (Evidence Partners).

The eligible population criteria included studies that examined patients/consumers, providers (both licensed and unlicensed), and/or population stakeholders (eg, payers, employers, communities, health systems, and the government) in outpatient care, ambulatory care, and long-term settings of primary care. Interventions had to target primary, secondary, tertiary, or quaternary prevention using at least one FDA/WHO approved or nonregulated digital health technology facet (eg, telehealth, mHealth, HIT, data analytics, and AI). No comparisons were required. Outcomes of interest included health (eg, individual- or population-level outcomes), health care performance (eg, as per the Agency for Healthcare Research and Quality [AHRQ]: access, quality, utilization, and efficiency, with measures categorized as structural, process, or outcomes including clinical/physiological, surrogate/intermediate, patient-centered, or patient-reported), and DHI implementation (eg, taxonomy as per Proctor et al: acceptability, adoption, appropriateness, costs, feasibility, fidelity, penetration, and sustainability) [15]. Only English-language primary studies or systematic reviews with the same inclusion criteria published between January 2014 and July 2020 were included. For definitions and descriptions of terms, see Multimedia Appendix 8 and Multimedia Appendix 9. Notable exclusion criteria for interventions included DHIs associated with treatment or diagnosis (except for preventive screenings), medical imaging for diagnosis, and telehealth using only noncellular telephone communication. Studies conducted in critical care (eg, intensive care unit) or inpatient (eg, hospital admission) settings were excluded.

### Data Extraction

After a series of data form piloting and discussions by all extractors to identify gaps in data extraction forms and ensure consistency in the application of definitions, data were abstracted into standardized forms within DistillerSR (Multimedia Appendix 10) for synthesis by a single reviewer. All fields of the data extraction forms for each article were examined for completeness by a second reviewer. Many data categorizations were not mutually exclusive, resulting in percentages totaling more than 100%.

### Subgroup Analysis and Data Synthesis

Following title and abstract screening, the large scope (>1000 titles) of the remaining included studies prohibited full-text review of all preventive DHIs identified globally. To narrow the scope of the geography and interventions under review, a subgroup analysis was performed; geography limits were set to only include studies conducted in the US. Additionally, it was apparent that a large volume of records focused on data analysis methods tangential to the development of DHIs. As such, studies that only used EHRs as a retrospective data capture tool were excluded. Two examples of excluded studies are a retrospective analysis of EHRs to determine the prevalence of a preventable disease and a study on the use of diagnostic telemedicine referral to a dermatologist.

Content analysis of extracted technology descriptions was performed to identify recurrent topics and more clearly understand the types of DHIs evaluated in the included studies according to *a priori* research questions in the protocol. This analysis yielded a list of articles selected to represent innovative or unique DHIs and their implementation in the final data set. Selected technologies were then narratively synthesized into 5 topical groups (eg, population-centered, patient-centered, care access expansion, panel-centered [dashboarding], and app-driven) to provide a framework for their analysis. Selected outcome (eg, health, health care performance, and implementation science) results from these articles were then extracted by a single reviewer to provide additional context regarding the impact of these DHIs beyond the directionality of their results. Details presented from this synthesis are not exhaustive, and key use cases have been highlighted in the results.

### Study Quality

Study quality was assessed using the Oxford levels of evidence [16], which allow for the categorization of evidence quality across heterogeneous study types. Examples of the study types comprising these evidence levels include (in increasing quality) expert opinion, case series, systematic reviews of case-control studies, individual cohort studies, randomized controlled trials (RCTs) with narrow confidence intervals, and systematic reviews of RCTs.

## Results

**Figure 1:**
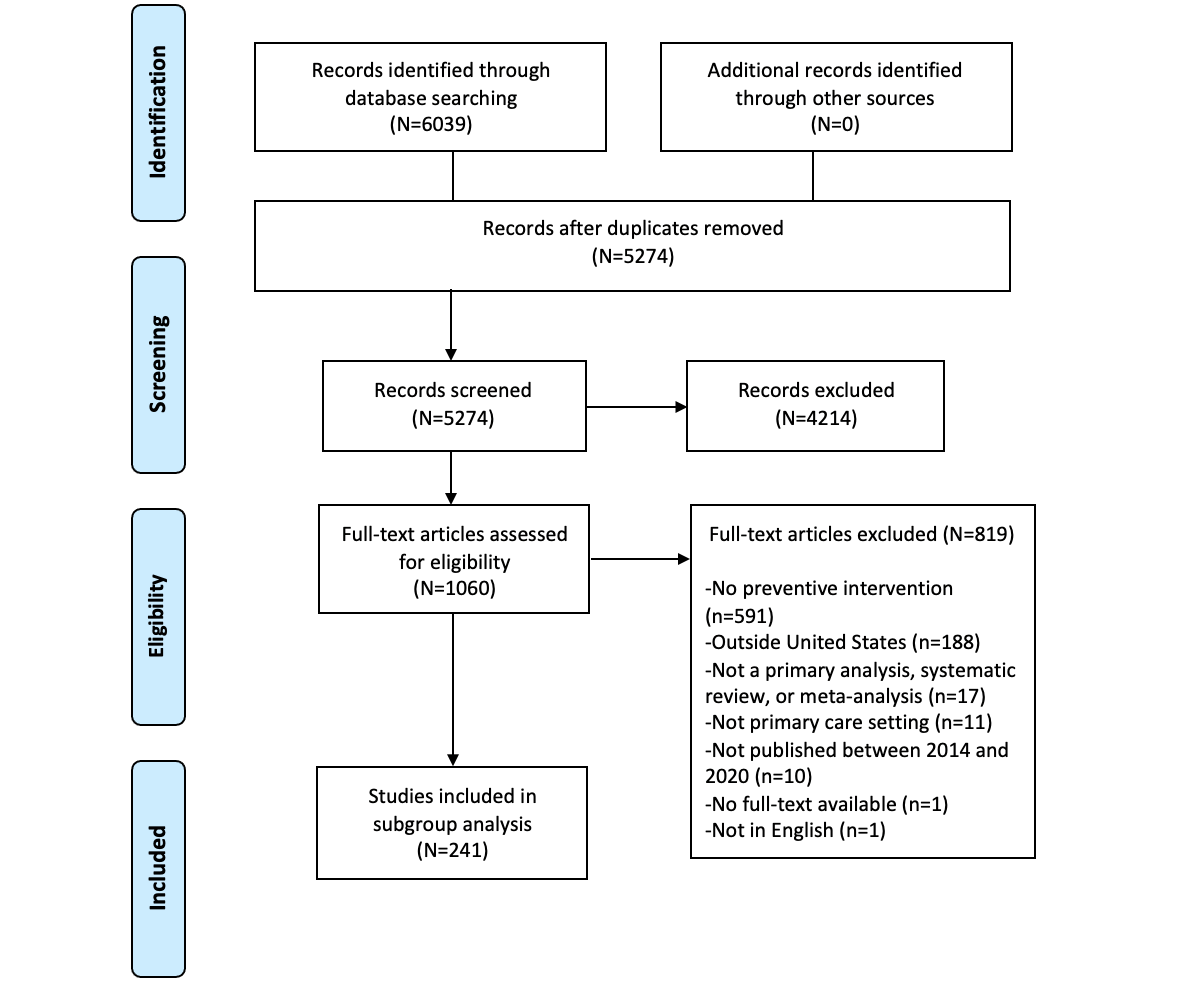
The flow diagram illustrates the flow of information through the different phases of the scoping review, including the number of records identified, included and excluded records, and the reasons for exclusion.

**Figure 2:**
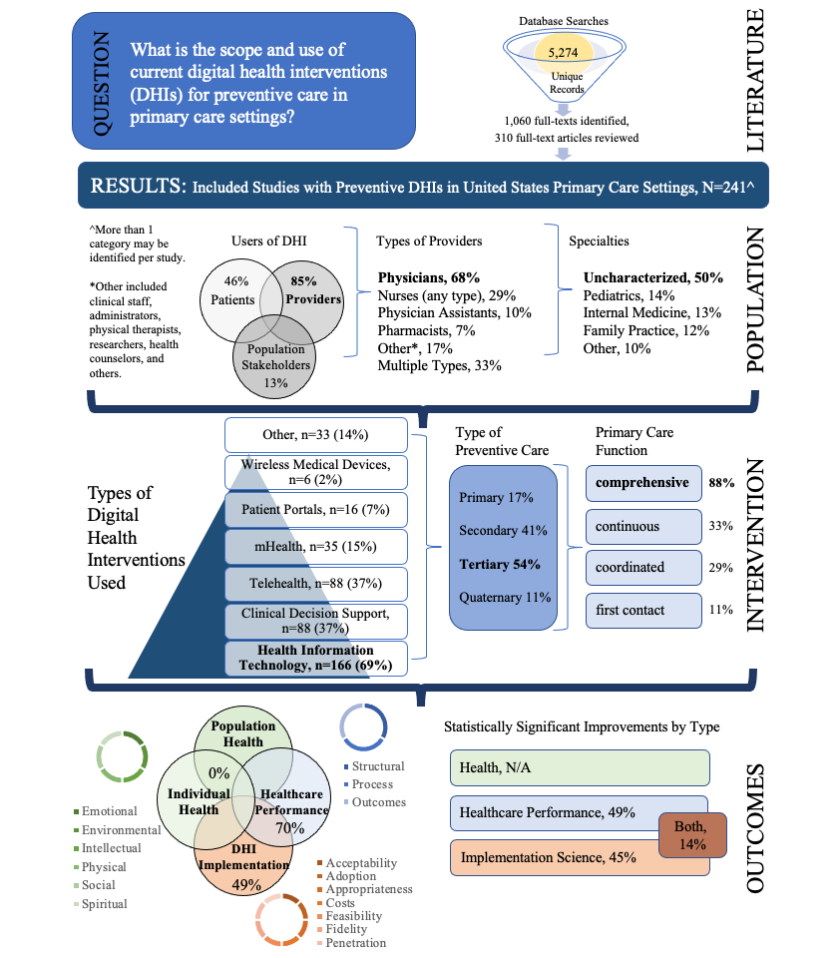
Summary of the study design and key findings. Scoping review study design and summarization of results across the categories of study population, intervention, and outcomes. N/A, not applicable.

Literature searches yielded 5274 unique citations, of which 1060 articles were eligible for full-text screening. A subgroup analysis was conducted to limit geography to US–only settings and exclude DHIs that evaluated EHRs as retrospective data capture tools. These applied limits resulted in 310 articles for full-text review, of which 241 articles [17-257] were included for the subgroup analysis (Figure 1). Abstractions of the included articles can be found in Multimedia Appendix 11. An overview of the study design and key findings is provided in Figure 2. The types of DHI articles covered included HIT (166/241, 68.9%), clinical decision support (88/241, 36.5%), telehealth (88/241, 36.5%), mHealth (35/241, 14.5%), patient portals (16/241, 6.6%), wireless medical devices (6/241, 2.5%), medical imaging (2/241, 0.8%), and other DHIs (31/241, 12.9%) (see Multimedia Appendix 9 for a description of each). The integration of multiple types of technologies was commonly applied to support DHIs (154/241, 63.9%) in practice. The most commonly identified combination of technology was the use of clinical decision support algorithms and mHealth to support more advanced care using HIT-related data.

The DHIs predominantly addressed tertiary prevention (131/241, 54.4%), followed by secondary (97/241, 40.3%), primary (40/241, 16.6%), and quaternary prevention (27/241, 11.2%), and a combination of prevention levels (43/241, 17.8%). The 4Cs primary care model by Dr Starfield was used as a framework to identify how DHIs supported delivery of preventive care; a large number of articles evaluated DHIs that demonstrated improvements in comprehensiveness of care (213/241, 88.4%), continuous care (76/241, 31.5%), coordinated care (69/241, 28.6%), and first contact care (26/241, 10.8%). The continuum of comprehensive care by DHIs included proactive anticipatory care, self-management support for patients, community resources for patients, longer patient visits to improve communication and clinician documentation, coding practices to improve accuracy, preventive care best practices (eg, immunizations, disease prevention and management, and reduction of overmedicalization), support for the increased scope of clinician practice, and knowledge-seeking practices.

DHI users were identified as providers (205/241, 85.1%), patients/consumers (111/241, 46.1%), others (31/241, 12.9%), or spanning multiple types (89/241, 36.9%). The types of providers using DHIs included physicians (163/241, 67.3%), nurses of any type (71/241, 29.5%), physician assistants (24/241, 10.0%), pharmacists (16/241, 6.6%), others (42/241, 17.4%), and multiple types (79/241, 32.8%). The “others” provider type included various clinic staff, administrators, technicians, physical therapists, researchers, health counselors, etc. The DHI user physician specialty characterization was as follows: uncharacterized (121/241, 50.2%), pediatrics (34/241, 14.1%), internal medicine (32/241, 13.2%), family practice (30/241, 12.4%), and others (25/241, 10.4%). Notably, 27 (11.2%) articles involved study settings with a mix of user types among majority Latino, African American, and Asian American populations, but only 6 (2%) of them discussed health disparities as the primary focus of their DHIs.

Primary and secondary outcomes for DHIs were predominantly clinical; 169 (70.1%) articles addressed clinical (eg, health care performance) outcomes, whereas 119 (49.4%) addressed nonclinical (eg, implementation science) outcomes. No identified studies examined health domain-related (eg, outcomes related to dimensions of wellness such as environmental, emotional, intellectual, physical, social, and spiritual) outcomes. A statistically significant improvement in relevant measured outcomes was identified in 192 (79.7%) articles, with 117 (48.5%) articles reporting improved health care performance outcomes (eg, preventive care/screening rates, validated tool scores, and medication adherence), 109 (45.2%) articles reporting improved implementation science outcomes (eg, intervention acceptability, adoption, and cost), and 34 (14.1%) articles reporting improvement in both. Among articles demonstrating statistically significant improvements in outcomes, 16 (6.6%) and 15 (6.2%) showed benefits for racial/ethnic groups specifically in health care and implementation science outcomes, respectively, with 4 (1.7%) articles identifying benefits for racial/ethnic groups in both. Moreover, 39 (16.2%) articles demonstrated only nonsignificant beneficial findings, while 8 (3.3%) articles provided no beneficial findings and only 1 (0.4%) article reported harm resulting from a DHI (in this case, limited to a portion of a subpopulation, whereas other populations received benefit).

Given that DHIs are frequently implemented as a combination of technologies, a content analysis was conducted to understand how DHIs identified in the included studies are collectively and uniquely being leveraged in care settings to impact prevention. Five topics were identified following content analysis that represent the most novel or distinct DHIs from the reviewed studies as follows: population-centered, patient-centered, care access expansion, panel-centered (dashboarding), and app-driven. Selected abstractions for the articles matching these topics are presented in Tables 1-5.

**Table 1:** Population-centered digital interventions for primary care.

First author, year	Study design	Description of technology	Sample size	Selected outcomes
Nagykaldi, 2014 [172]	Pre-post	Linking of a regional health system, hospital organization, and preventive services reminder system via HIE^a^.	346 patients (20% ethnic minorities)	12%-36% increase in preventive service documentation and delivery (*P*<.001). 9.6% increase in medication reconciliation (*P*<.001).
Nagykaldi, 2017 [171]	Pre-post	Wellness coordinator connection to HIE organizations, PCPs^b^, county health departments, and hospitals for preventive care outreach for rural communities.	9138 rural patients	3%-215% increase in delivery of 10 preventive services over 12 months (*P*=.004). 80% ROI^c^ for selective preventive services (range, 32%-122%). 40% ROI on wellness coordinator employment cost.
Fanizza, 2018 [81]	Open label nonrandomized	Pharmacist connection to the state HIE for comprehensive medication review after discharge and communication with prescribers.	40 patients	25.2% decrease in overall 30-day readmission rates (*P*=.03). 22.7% decrease in 30-day readmission rates for initial diagnosis (*P*=.009).
Shade, 2015 [199]	Pre-post	Clinic link to the state surveillance system providing alerts when out-of-care HIV patients present in the ED^d^ or other settings.	6 sites serving underserved communities	OR^e^ 2.61 (95% CI 2.11-3.21) for care retention (*P*=.001). OR 1.24 (95% CI 1.03-1.49) for being on ART^f^ (*P*=.02). OR 4.16 (95% CI 2.54-6.80) for undetectable viral load (*P*<.001).

^a^HIE: health information exchange.

^b^PCP: primary care provider.

^c^ROI: return on investment.

^d^ED: emergency department.

^e^OR: odds ratio.

^f^ART: antiretroviral therapy.

**Table 2:** Selected patient-centered digital health interventions for primary care (direct engagement).

First author, year	Study design	Description of technology	Sample size	Selected outcomes
Grant, 2015 [97]	RCT^a^	Informatics surveillance and reminder system connected to EHR^b^ lab test orders that generates mailed letters requesting patient completion of labs for hyperlipidemia, diabetes, and HTN^c^ monitoring.	4038 patients	aHR^d^ 1.26 (95% CI 0.99-1.62) for decreased time to LDL^e^ goal. aHR 1.15 (95% CI 1.01-1.32) for earlier LDL lab assessment.
Hess, 2014 [110]	Observational cohort	PHR^f^ delivering active notifications regarding gaps in preventive chronic disease monitoring until patient logs on to the PHR or closes the prevention gap.	584 patients	58% of all prevention gaps were closed over 12 months. 61% of notified patients accessed the PHR or closed the triggering care gap after the 1st message and 73% after the 2nd message.
Hojat, 2020 [112]	Controlled trial	EHR bulk-ordered HCV^g^ antibody testing plus automatic PHR messages requesting patients to go to the lab.	1024 patients	14% increase in completed HCV tests (*P*<.001; OR^h^ 1.7, 95% CI 1.2-2.1). Only 3.5% of patients responded to PHR messages, and repeat messaging had no effect on completion.
Langford, 2019 [138]	Observational cohort	SMS text message contact to help underserved patients with diabetes find their optimal basal insulin dose.	113 patients	84% of patients reached optimal insulin dose. Age, copay status, and initial fasting blood glucose were significantly associated with 100% SMS response (*P*≤.03).
Mehta, 2018 [163]	RCT	Patient portal message containing either opt-in or opt-out for FIT^i^ colorectal cancer screening test.	127 patients	28% higher FIT completion rate for patients receiving opt-out messages.
Quanbeck 2018 [185]	Observational cohort	Patient discussion board, interactive modules for health tracking, and self-management and coping with cravings for addiction management. Clinician web portal for patient-generated data.	268 patients	44% reduction in risky drinking days (*P*=.04), and 34% reduction in illicit drug use days (*P*=.01), over 12 months. 53%-60% of patients accessed the intervention during the final week of the implementation period.
Smallwood, 2017 [211]	RCT	Patient portal decision support tool for fracture risk and prevention. Includes educational information, risk calculation, and a treatment decision values elicitation exercise.	50 patients	Improved decision quality (*P*<.001) and conflict (*P*<.001) scores after the intervention. 25.7% (*P*=.046) increase in treatment decisions 3 months after the intervention.
Turvey, 2016 [234]	RCT	Patient portal link to a downloadable and printable CCD^j^ for sharing with non-VA^k^ providers for continuity of care.	52 patients	73% increase in the proportion of patients sharing the CCD with non-VA providers with training on accessing the CCD (*P*<.001). No improvement in medication reconciliations, but significant reduction in duplicate laboratory tests ordered by non-VA providers (*P*=.02).
Woo, 2016 [241]	Pilot	Daily customized spinal cord injury/disorder disease management questions delivered to patients via a data messaging device. Provider web portal with patient responses and risk level ratings.	33 patients	Average total response rate of 56%, ranging from 10% to 93%. Nearly 20% decrease in the DUSOI^l^ score over 6 months.
Yakovchenko, 2019 [245]	RCT	Customized SMS reminder messages about HCV treatment appointments, labs, adherence, and motivation.	71 patients	Lower distress about failing treatment (*P*=.05) and better medication adherence (*P*=.06). 96% of texters vs 94% of nontexters achieved SVR^m^.

^a^RCT: randomized controlled trial.

^b^EHR: electronic health record.

^c^HTN: hypertension.

^d^aHR: adjusted hazard ratio.

^e^LDL: low-density lipoprotein.

^f^PHR: personalized health record.

^g^HCV: hepatitis C virus.

^h^OR: odds ratio.

^i^FIT: fecal immunochemical test.

^j^CCD: continuity of care document.

^k^VA: Veterans Affairs.

^l^DUSOI: Duke Severity of Illness Checklist.

^m^SVR: sustained virologic response.

**Table 3:** Selected care access expansion digital health interventions (virtual care/telehealth).

First author, year	Study design	Description of technology	Sample size	Selected outcomes
Aikens, 2015 [20]	Observational cohort	Weekly IVR^a^ calls for depression self-management. Option to designate a lay support person to receive email reports summarizing reported symptoms and providing problem-tailored support guidance.	221 patients	Increases of 20% in the per-week aOR^b^ for medication adherence and 16% for depression remission compared with controls.
Coker, 2019 [58]	RCT^c^	Telehealth-enhanced referral to a CMHC^d^ using informational videos, SMS text messages, and telehealth screening at the primary care clinic.	342 Latino children	aOR 3.02 (95% CI 1.47-6.22) for completing CMHC visits compared with controls. Telehealth referrals took longer to complete screening but reported greater satisfaction with referral than controls.
Halterman, 2018 [103]	RCT	Videoconference telemedicine visit in a school health office for asthma baseline and medication; follow-up telemedicine assessments every 4-6 weeks.	400 urban students	0.69 (95% CI 0.15-1.22) more symptom-free days per 2 weeks (*P*=.01). aOR 0.52 (95% CI 0.32-0.84) for asthma-related ED^e^ visit or hospitalization.
Osofsky, 2017 [178]	Pre-experimental time series	Onsite and/or telemedicine behavioral-based trauma treatment delivered in primary care clinics.	235 patients	4.5-point decrease in the PCL-C^f^ score (*P*=.001), and 1.8-point decrease in the PHQ-15^g^ score (*P*=.001).
Perry, 2018 [181]	RCT	Live video telemedicine asthma education at school for a child, caregiver(s), and school nurse; telemonitoring of patient-reported symptoms; PCP^h^ prompts with guideline-based asthma management.	393 rural African American students	No change in symptom-free days, quality of life, or lung function. 42% increase in peak flow meter use compared with controls (*P*<.01) and 19% increase in medication adherence (*P*=.03) over 6 months.
Reeves, 2016 [186]	Pre-post	Implementation of EHRs^i^ in the school system for the asthma care program; messaging connection to PCP EHR systems; school nurse asthma template for PCP messaging.	33 students	39.4% decrease in asthma inpatient admissions (*P*<.001) and 18.2% decrease in exacerbations (*P*<.05) over 12 months.
Richter, 2015 [189]	RCT	Live video telehealth for tobacco cessation delivered in primary care clinics.	566 patients	No difference in biochemically verified prevalence, prolonged abstinence, quit attempts, or number of cigarettes smoked per day compared with phone counseling.

^a^IVR: interactive voice response.

^b^aOR: adjusted odds ratio.

^c^RCT: randomized controlled trial.

^d^CMHC: community mental health clinic.

^e^ED: emergency department.

^f^PCL-C: posttraumatic stress disorder checklist-civilian version.

^g^PHQ-15: 15-item patient health questionnaire.

^h^PCP: primary care physician.

^i^EHR: electronic health record.

**Table 4:** Panel-centered digital health interventions for primary care (dashboarding).

First author, year	Study design	Description of technology	Sample size	Selected outcomes
Allen, 2017 [24]	RCT^a^	Culturally sensitive team model using an electronic diabetes dashboard providing alerts and reports for each patient regarding clinical and behavioral factors and social distress.	399 Latino patients	Social distress score decrease of 0.6 (controls) vs 1.6 (intervention) over 6 months (*P*=.01).
Duquaine, 2015 [75]	Observational cohort	CDS^b^ for tobacco use and interventions for smoking cessation; quarterly communications with practice-specific and overall program performance.	19 clinics treating low-income and Medicaid patients	Successful implementation at all sites. Change in EHR^c^ documentation of prevalence and cessation rates (NR^d^).
Fiks, 2015 [85]	Open-label nonrandomized	Quarterly feedback reports summarizing personal, practice, and network rates of missed HPV^e^ vaccine opportunities.	227 PCPs^f^	5.7% (95% CI 3.8-7.7) increase in HPV vaccination compared with controls.
Kapoor, 2018 [127]	Observational cohort	Emailed report of the proportion of atrial fibrillation patients receiving anticoagulation therapy compared to peers plus EHR message 1 day before visits with anticoagulation eligible patients.	5406 patients	Providers reviewed emails (45%) and EHR messages (96%), demonstrating feasibility. No change in the percentage of patients receiving anticoagulation therapy compared with controls after 3 months.
Zimmerman, 2017 [255]; Nowalk, 2016 [174]; Zimmerman, 2017 [257]; Lin, 2016 [145]; Zimmerman, 2017 [256]	RCT and pre-post	4 Pillars Immunization Toolkit and Practice Transformation Program. Web-based dashboard providing and tracking strategies for increasing practice vaccination rates, including EHR prompts, digital outreach, and standing order programs.	25 clinics [255]; 24 clinics [174]; 11 clinics [257]; 25 clinics [145]; 22 clinics [256]	2.7% to 10.2% statistically significant increases in vaccination rates for intervention and control sites during RCT studies. −1.9% to 17.1% statistically significant increases in vaccination rates for active intervention groups during year 2 of the pre-post study.

^a^RCT: randomized controlled trial.

^b^CDS: clinical decision support.

^c^EHR: electronic health record.

^d^NR: not reported.

^e^HPV: human papillomavirus.

^f^PCP: primary care physician.

**Table 5:** Selected app-driven digital health interventions for primary care.

First author, year	Study design	Description of technology	Sample size	Selected outcomes
Bennett, 2018 [35]	RCT^a^	App using IVR^b^ and SMS text messaging to collect patient behavior change data and weight via a smart scale, provide tailored patient feedback based on goal progression, and generate EHR^c^ counseling recommendations for clinicians.	351 patients	−4.4 kg (95% CI −5.5 to −3.3) weight loss at 6 months (*P*<.001); −3.8 kg (95% CI −5.0 to −2.5) weight loss at 12 months (*P*<.001). Participants completing ≥80% of interactions lost significantly more weight than less engaged participants (*P*<.01).
Brayboy, 2016 [45]	Pre-post	iPhone-compatible app for providing trusted, age-appropriate, straightforward sexual health information and resources to teenage girls.	20 teenage girls	3.4%-4.2% improvement in sexual health topic knowledge. 58.8% increase in the perception that they or other teenage girls would use the app (*P*<.001).
Dahne, 2019 [64]	RCT	Self-help app adaptation of Brief Behavioral Apptivation, including education, identification of values, daily mood monitoring, and social support including gamification, to reinforce continued use.	52 patients	63% greater decrease on BDI-II^d^ assessment after treatment compared with usual care. 70% of participants continued to use the app 1 month after enrollment, and 50% continued to use it at 2 months.
Gustafson, 2014 [99]	RCT	Smartphone app to support alcoholism recovery using alerts for trigger locations, audio-guided relaxation, PRO^e^ measurement, and clinician notification, as well as a panic button for contacting support persons.	349 patients	1.37 (95% CI 0.46-2.27) fewer risky drinking days than controls over 12 months (*P*=.003). OR 1.65 (95% CI 1.05-2.57) for abstinence prevalence over 12 months (*P*=.03).
Leddy, 2019 [140]	RCT	Home smartphone urinalysis test to complete proteinuria screening for HTN^f^ management. SMS text message link for downloading the app, obtaining the home testing kit, and receiving PCP^g^ notification of abnormal results.	999 patients	10.9% increase in proteinuria screening completion (*P*<.001). 89% of home test patients preferred home testing over a visit to the physician’s office.
Lv, 2017 [149]	Pre-post	Dashboard of patient’s personalized action plan, treatment goals, and self-monitoring data combined with a wireless BP^h^ monitor, smartphone, study app, pedometer, and web messaging system.	147 patients	55.9% increase in the proportion of patients meeting office BP goals (<140/90 mmHg) at 6 months (*P*<.001). 46.2% increase in the proportion of patients meeting home BP goals (<135/85 mmHg) at 6 months (*P*<.001).
Ofili, 2018 [176]	Pre-post	App with diabetes curriculum, goal identification and tracking, connectivity to consumer devices (eg, activity monitors), and health coach consultation.	287 patients	Improvements in SBP^i^ (6 mmHg), blood glucose (15 mg/dL), and physical activity (0.56 miles/day) at 12 weeks (all *P*<.01), which continued through 52 weeks.
Yu, 2018 [249]	Pre-post	App delivering a guided cognitive behavioral program for generalized anxiety disorder along with in-app coach pairing and messaging.	63 patients	3.6-point mean reduction on GAD-7^j^ over 2 months for patients with baseline GAD-7 ≥8 (*P*<.001).

^a^RCT: randomized controlled trial.

^b^IVR: interactive voice response.

^c^EHR: electronic health record.

^d^BDI-II: Beck Depression Inventory II.

^e^PRO: patient-reported outcome.

^f^HTN: hypertension.

^g^PCP: primary care provider.

^h^BP: blood pressure.

^i^SBP: systolic blood pressure.

^j^GAD-7: Generalized Anxiety Disorder-7.

Primary prevention targets focused on the use of population-centered [171,172] and panel-centered [85,145,175,255-257] DHIs to improve adolescent [256,257] or adult [145,171,172,174,255] vaccination rates for human papillomavirus [256,257], influenza [145,171,172,257], pneumococcal disease [171,172,255,257], and Tdap (tetanus, diphtheria, and pertussis) [174,257].

All the above DHIs that targeted primary prevention had statistically significant health care [145,172,255-257] or implementation [171,174] outcomes following the intervention. Identifying return on investment (ROI) and value on investment can be large barriers for DHI implementation; however, both were satisfied when a community wellness registry was connected to EHRs via a health information exchange (HIE). This pilot study demonstrated the feasibility and cost-effectiveness of technology implementation in a community-based model with a mean ROI of 80% (range, 32% to 122%) for the improved delivery of 10 selective preventive services (mean increase 35%, range 3% to 215%; *P*=.04) in rural settings [171].

Patient-centered [112,163,211] and population-centered [171,172] DHIs supported secondary prevention by examining measures that led to early diagnosis and treatment using direct-to-patient messaging in an EHR [112,163], decision aids embedded in patient portals [211], and an intelligent HIE using clinical decision support [171,172]. These DHIs improved screening rates for cancer (eg, breast [172] and colorectal [163,171,172]), hepatitis C virus (HCV) [112], and osteoporosis [171,211]. Only 1 study in this grouping did not have significant improvements following the DHI, which may be due to the more invasive and costly colonoscopy procedure itself rather than the ineffectiveness of the EHR portal messaging intervention to improve colorectal cancer screening [163]. However, an advanced EHR that used population analytics and bulk laboratory ordering to directly engage patients for universal HCV screening nearly doubled testing (odds ratio 1.7, 95% CI 1.2-2.1) in the intervention group [112].

Tertiary prevention for chronic disease management was supported primarily by care access expansion [20,58,103,178,181,189], app-driven [35,64,140,149,176,249], and patient-centered [38,97,241,245] approaches. Overall, DHIs decreased disease severity and associated comorbidities; lowered the numbers of emergency department visits, hospitalizations, and 30-day readmissions; increased the receipt of follow-up care; improved medication adherence in the identified studies [20,35,58,64,97,103,149,176,199,245,249]; and improved the quality or effectiveness of health services by technology implementation [24,38,103,140,171,178,186,189,249]. Disease areas targeted by DHIs included diabetes [38,97,171,176], hypertension [97,140,149], asthma [103,181,186], obesity [35,171], cardiovascular disease [123], HIV [199], HCV [245], and hyperlipidemia [97]. Management of behavioral health included smoking cessation support [75,171,189], promotion of physical activity [171], substance abuse management [99,185], and sexual health education [45] using mHealth technology. Mental health [58] (eg, depression [20,64], anxiety [249], posttraumatic stress disorder [178], and social distress [24]) improved following digital interventions. Notably, telehealth and mHealth were leveraged predominantly to support mental health interventions with care access expansion [20,58,178] and app-driven [64,249] technologies to improve patient function, minimize illness impacts, and decrease associated complications. Behavioral and mental health conditions and other chronic diseases often occur concurrently [258]. Two studies [178,185] integrated behavioral or mental health DHIs for chronic condition care, but only 1 study [178] reported outcome measures for both mental and physical health, whereby both improved significantly.

Notably, none of the studies identified by the content analysis examined DHIs for quaternary prevention.

Using the Oxford levels of evidence [16], the quality of the included studies was moderate to low overall due to many studies (101/241, 41.9%) presenting level 4 evidence (eg, case series, poor quality cohort, and case-control studies) and the remainder displaying level 1b (eg, individual RCTs with a narrow CI; 46/241, 19.1%), 2b (eg, individual cohorts including low-quality RCTs; 58/241, 24.1%), 2c (eg, outcomes research and ecological studies; 17/241, 7.1%), 3b (eg, individual case-control studies; 17/241, 7.1%), or 5 (eg, expert opinions; 3/241, 1.2%) evidence.

## Discussion

### Principal Findings

Amidst the rapid digital transformation of the primary care delivery system in response to the COVID-19 pandemic, this is the first comprehensive summary on DHIs in use by interdisciplinary clinicians (eg, physicians, pharmacists, psychiatrists, etc) in primary care. This scoping review and its subgroup analysis summarized a growing evidence base and rendered a collection of potentially successful strategies for patients, providers, and population stakeholders to improve outcomes for health, health care performance, and implementation science through the use of DHIs. Moreover, important scientific gaps were identified in the contemporary evaluation and knowledge of DHIs leveraged in primary care, particularly the scarcity of the evaluation of DHIs in health disparities and evaluation of the negative effects of DHIs.

A few major themes emerged from our analysis of the extracted data. First, the digital health technologies identified and reviewed were highly concentrated in a narrow range of HIT, most specifically around EHRs/electronic medical records, particularly with the use of alerts to help clinicians make appropriate clinical decisions. Though understandable given their high use and decade-long attention to increasing adoption via “meaningful use” in primary care [259,260], the absence of DHI literature involving other platforms was telling. Despite unprecedented attention to telehealth implementation due to the COVID-19 pandemic response, little evidence of effective implementation of this specific DHI exists to guide primary care telehealth use for health care delivery in the US. A few studies did examine more innovative uses of technology, particularly for the delivery of mental and behavioral health (Tables 1-5). As HIT continues to rapidly evolve and health care is delivered in more innovative ways due to the COVID-19 pandemic, more research should focus on novel DHIs applied to primary care.

Second, despite prevention being 1 of 6 mechanisms underpinning primary care’s beneficial impact on population health [261] and an early target for DHIs, studies evaluating prevention were predominantly focused on secondary or tertiary preventive interventions. Most would agree that disease prevention offers the greatest yield for population health and is amenable to DHIs via mobile and online apps, clinical kiosks, and electronic patient portals [262,263]. Primary prevention interventions, such as immunizations, rely on effective patient counseling and education, which can be difficult and time-consuming to document and capture in EHRs (the predominant type of intervention found in our review). This finding may be a reflection of physician roles in the US. Traditionally, the role of primary prevention has relied on public health professionals [264], and although primary care physicians are increasing their ability to address the needs of the community, most physicians are still focused on the needs of the individual [265]. As the intersection of public health and primary care becomes more urgent to strategically improve individual and population health, future studies should examine the role of DHI adoption and implementation in their integration.

Third, DHIs enhanced core primary care functions by contributing to the comprehensiveness of care provided. This was an unexpected finding given that DHIs are often thought of in the context of first contact through patient portals, coordination through electronic referrals and linked EHRs, and continuity through HIEs and sharing of documents. Many of the articles reviewed discussed the use of DHIs to identify patients in need of services and alert clinicians to provide them. For example, multiple studies described EHR alerts that would prompt clinicians to order viral hepatitis C testing for patients with indications for screening (Multimedia Appendix 11) [83,93,112,116,133,150,164,173,207,246]. Other studies shared examples of how patients could be trained to provide services for themselves (Table 2) or how DHIs could be used to offer additional clinical services (Table 3). Thus, it makes sense that comprehensiveness, or the provision of a robust set of services to a patient, would be improved with DHIs. In an era where comprehensiveness of care is said to be declining in primary care [266-271], DHIs may provide an innovative solution for primary care practices to increase and enhance the services they provide.

Finally, while the development and release of health apps continue to increase, few evaluations of app-driven DHIs were identified in our study (Table 5). This may be in part because many apps lacked integration with primary care or other technology systems or because of the evolving standards for evaluating these types of interventions, as evidenced by the recent establishment of the FDA’s Digital Health Center of Excellence [272]. Most app-driven DHIs included in our study were patient-facing and focused on helping to better involve patients in their care. However, app-driven DHIs are also capable of providing an overwhelming amount of data to providers. Balancing data collection features from apps by adding functionalities, such as thresholds triggering clinical alerts/feedback, designing patient-counseling suggestions based on gathered data, and pairing with timely coaching/contact is important to enhance the clinical relevance and quality of these tools. As the development and clinical adoption of app-driven DHIs continue to expand, rigorous investigation of their safety, efficacy, and value in primary care is urgently needed.

### Limitations

These results should be interpreted in the context of a few limitations. The findings are limited to studies conducted in US settings, which prohibits the generalization of their applicability and use at a global scale. Review of the use of DHIs in non-US primary care settings should be prioritized in future work. Further, due to the heterogeneity of identified interventions, it is not possible to provide head-to-head comparisons. The large heterogeneity of DHIs is an additional reason why our synthesis focused on the novel and distinct DHIs that are collectively used in primary care practice rather than presenting evidence collated by distinct DHI technologies. Other limitations include single screening of titles and abstracts, English language restriction, and lack of gray literature evaluation. Data extraction for each article was not confirmed by a secondary reviewer, leaving room for bias in the interpretation of the articles. For example, it was left up to each reviewer to determine the type of prevention the DHI was addressing, or which primary care function (eg, the 4Cs by Dr Starfield: first contact, comprehensiveness, continuity, and coordination) the DHI enhanced. However, careful and collaborative definition of our processes and outcomes prior to extraction (ie, types of prevention or primary care functions) should minimize this bias. Lastly, we intentionally selected a quality assessment tool rather than a risk of bias tool, as we only planned to measure the extent that methodological safeguards (ie, internal validity) against bias were implemented. A risk of bias assessment would have offered a bias judgement (ie, estimation of intervention effects) on such a quality assessment, and judgement of the evidence may have shifted with this approach. It is important to consider that even when a study implements all possible safeguards in a tool, it may not be unbiased, and conversely, a study applying no safeguards is not necessarily biased [273].

### Conclusions

Gayle Stephens noted in 1965 that “One of the paradoxes of our time is that the healing relationship seems most in jeopardy at a time when we need it most,” commenting on the range of “forces which threaten to depersonalize the meeting of a doctor and patient” [274]. That paradox remains in an age where technology is often seen as distracting rather than enhancing care. Through further adoption of DHIs with evidence of effectiveness, providers and patients/consumers can enhance primary care by improving the delivery of preventive services and promoting more comprehensive care. Yet, relying solely on EHR alerts may not lead to substantial improvements in health care in the US. Moreover, rigorous and prospective evaluations of the potential negative effects of these DHIs, particularly for clinical end users of these technologies, will be needed to ensure holistic improvement of health care. Innovative DHIs should undergo evaluation in well-designed studies to generate evidence and establish best practices that can be replicated and scaled in diverse primary care settings. Given the ability of technology to amplify existing health disparities and biases, the development of DHIs that can help overcome health disparities and the evaluation of the benefits and harms of current DHIs on health disparities are imperative. In addition, DHIs that allow integration of public health with primary care will be essential for rapid and effective responses to health and health care challenges, such as the COVID-19 pandemic, in an increasingly technology-driven health care environment.

## Appendix

Multimedia Appendix 1 Search outline using the PCC (participants, concept, and context) framework.

Multimedia Appendix 2 MEDLINE search via PubMed.

Multimedia Appendix 3 Cochrane Library search.

Multimedia Appendix 4 Embase search.

Multimedia Appendix 5 Updated MEDLINE search via PubMed.

Multimedia Appendix 6 Updated Cochrane Library search.

Multimedia Appendix 7 Updated Embase search.

Multimedia Appendix 8 Inclusion/exclusion criteria.

Multimedia Appendix 9 Description of digital health intervention categories.

Multimedia Appendix 10 Data extraction forms.

Multimedia Appendix 11 Abstracted results from included articles.

## Abbreviations

AI: artificial intelligence
DHI: digital health intervention
EHR: electronic health record
FDA: Food and Drug Administration
HCV: hepatitis C virus
HIE: health information exchange
HIT: health information technology
mHealth: mobile health
RCT: randomized controlled trial
ROI: return on investment
WHO: World Health Organization
