# Supplementary material for: Digital Health Interventions to Enhance Prevention in Primary Care: Scoping Review
Source: JMIR Med Inform. 2022 Jan 21;10(1):e33518. doi: 10.2196/33518 (PMC8817213; doi:10.2196/33518)
Supplement: Multimedia Appendix 1 [file medinform_v10i1e33518_app1.docx]

**Multimedia Appendix 1.** Search outline using the PCC (participants, concept, and context) framework.

| PCC | Term | Keywords | MeSH |
| --- | --- | --- | --- |
| Participants | General terms for primary care | primary care | primary health care; physicians, primary care |
|  | Primary care specialty – family practice | family practice, family medicine, general medicine | family practice; physicians, family; general medicine |
|  | Primary care specialty – internal medicine | internal medicine AND primary care; general internal medicine AND primary care | internal medicine AND primary care |
|  | Primary care specialty – pediatrics | pediatric, pediatrician, general pediatricians | pediatrics; pediatricians |
|  | Primary care specialty – Internal Medicine & Pediatrics | (med-pedes, med/pedes, med pedes, med peds, med-peds, med/peds), AND primary care |  |
|  | Primary care specialty - geriatrics | geriatric, geriatrician | geriatrics; geriatricians |
|  | Non-physician primary care professionals | (nurse practitioner, physician assistant, pharmacist, PharmD) AND primary care | (nurse practitioners; physician assistants; pharmacists) AND primary care |
| Concept | Digital health defined by FDA/WHO | electronic health record, personal health record, electronic medical record, EMR, EHR,  computer security, data security, cybersecurity, cyber security, data protection, data encryption,  cloud computing, cloud process, cognitive computing  patient web portal, patient web-portal, patient portal, web portal  mobile technology, telemedicine, telehealth, mobile health, mHealth, eHealth, m-Health, mobile-health, telecommunication, app or application within use of smartphone, smart-phone, mobile, or phone  clinical decision support, decision support system  health information exchange, electronic health information, electronic health communication, interoperability,  patient monitor, wearables, activity monitor, sensor, physiologic monitoring | electronic health records; health records, personal;  computer security;  cloud computing;  patient portals;  telemedicine;  decision support systems, clinical;  health information exchange;  health information interoperability;  physiologic monitoring; |
|  | Digital health not defined by FDA/WHO | artificial intelligence, machine intelligence, computational intelligence, machine learning, natural language processing, neural network, quantified self, connected health, big data, gamification, social media, health 2.0, personalized genomics, precision medicine, individualized medicine | artificial intelligence;  machine learning; precision medicine |
| Context | Patient Care Management in Preventive Medicine | prevention, preventive, mass screening, screening, care management, comprehensive care, care planning, disease/care management | prevention and control, mass screening, preventive health services, patient care management |
