# Supplementary material for: Digital Health Interventions to Enhance Prevention in Primary Care: Scoping Review
Source: JMIR Med Inform. 2022 Jan 21;10(1):e33518. doi: 10.2196/33518 (PMC8817213; doi:10.2196/33518)
Supplement: Multimedia Appendix 3 [file medinform_v10i1e33518_app3.docx]

**Multimedia Appendix 3.** Cochrane Library search.

| **Search no.** | **Facet** | **Search Terms** | **Search Results**  **(October 15, 2019** |
| --- | --- | --- | --- |
| 1 | Primary care | [mh “primary health care”] OR [mh "Physicians, Primary Care"] OR “primary care”:ti,ab | 19,685 |
| 2 | Primary care specialty – family practice | [mh "Family Practice"] OR “family practice”:ti,ab OR [mh "Physicians, Family"] OR "family physician*":ti,ab OR “family medicine”:ti,ab OR [mh “general medicine”] OR “general medicine”:ti,ab | 3,740 |
| 3 | Primary care specialty – internal medicine | ([mh "Internal Medicine"] OR “internal medicine”:ti,ab) AND “primary care”:ti,ab | 298 |
| 4 | Primary care specialty – pediatrics | [mh "Pediatrics"] OR pediatric*:ti,ab OR [mh "Pediatricians"] OR pediatrician*:ti,ab | 28,320 |
| 5 | Primary care specialty - geriatrics | [mh "Geriatrics"] OR “geriatric*”:ti,ab OR [mh "Geriatricians"] OR “geriatricians”:ti,ab | 5,111 |
| 6 | Non-physician primary care professionals | ([mh "Nurse Practitioners"] OR “nurse*”:ti,ab OR “nurse practitioner*”:ti,ab OR [mh “Physician Assistants”] OR “physician assistant*”:ti,ab OR [mh "Pharmacists"] OR “pharmacist*”:ti,ab) AND (“primary care”:ti,ab OR “family practice”:ti,ab OR “family medicine”:ti,ab OR “general medicine”:ti,ab) | 2,069 |
| 7 | All  primary care professionals | #1 OR #2 OR #3 OR #4 OR #5 OR #6 | 54,203 |
| 8 | Digital health and health behavior technologies synonyms | "digital health":ti,ab OR "digital health intervention":ti,ab OR "digital behavior change":ti,ab OR "digital behaviour change":ti,ab OR "digital health technology":ti,ab | 147 |
| 9 | Specific types of  digital health technologies outlined by FDA/WHO | [mh  “Electronic Health Records”] OR “electronic health record*”:ti,ab OR personal health record*:ti,ab OR electronic medical record*:ti,ab OR “EMR”:ti,ab OR “EHR”:ti,ab OR [mh "Health Records, Personal"] OR    [mh "Computer Security"] OR "data security":ti,ab OR "cybersecurity":ti,ab OR "cyber security":ti,ab OR “data protect*”:ti,ab OR “data encrypt*”:ti,ab OR    [mh "Cloud Computing"] OR "cloud computing":ti,ab OR "cloud process*":ti,ab OR cognitive comput*:ti,ab OR    [mh "Patient Portals"] OR patient web portal*:ti,ab OR patient web-portal*:ti,ab OR patient portal*:ti,ab OR web portal*:ti,ab OR    mobile technolog*:ti,ab OR [mh "Telemedicine"] OR "telemedicine":ti,ab OR telehealth*:ti,ab OR "mobile health":ti,ab OR "mHealth":ti,ab OR "eHealth":ti,ab OR "m-Health":ti,ab OR "mobile-health":ti,ab OR telecommunication*:ti,ab OR ((app OR application*) near/3 (smartphone* or smart-phone or mobile* or phone*)) OR     [mh "Decision Support Systems, Clinical"] OR "clinical decision support":ti,ab OR "decision support system":ti,ab OR    [mh "Health Information Exchange"] OR health information exchange*:ti,ab OR “electronic health information”:ti,ab OR electronic health communication*:ti,ab OR [mh “health information interoperability”] OR “interoperability”:ti,ab OR    patient monitor*:ti,ab OR “wearables”:ti,ab OR “activity monitor*”:ti,ab OR sensor*:ti,ab OR [mh “physiologic monitoring”] | 25,347 |
| 10 | Specific types of  digital health technologies not outlined by the FDA/WHO but are of interest | [mh "Artificial Intelligence"] OR "artificial intelligence":ti,ab OR "machine intelligence":ti,ab OR "computational intelligence":ti,ab OR [mh "Machine Learning"] OR  "machine learning":ti,ab OR "machine-learning":ti,ab OR “natural language processing”:ti,ab OR neural network*:ti,ab OR “quantified self”:ti,ab OR “connected health”:ti,ab OR "big data":ti,ab OR "gamification":ti,ab OR "social media":ti,ab OR "health 2.0":ti,ab OR "personalized genomics":ti,ab OR "precision medicine":ti,ab OR [mh "precision medicine"] OR "individualized medicine":ti,ab OR “internet of things”:ti,ab OR “IoT”:ti,ab OR “IOT”:ti,ab    OR ((“social programs” OR care manage*:ti,ab OR coordinate* care:ti,ab or health benefit*:ti,ab or insur*:ti,ab) AND “digital”:ti,ab) | 3,896 |
| 11 | Combine digital health technology  strings | #8 OR #9 OR #10 | 28,864 |
| 12 | Combine digital health technology with primary care strings | #7 AND #11 | 2,917 |
| 13 | Identify prevention and control subheading | [mh/PC] | 89,179 |
| 14 | Prevention and care management studies using digital health in primary care | #12 AND (#13 OR “prevention”:ti,ab OR “preventive”:ti,ab OR [mH “mass screening”] OR “screening”:ti,ab OR [mh “preventive health services”]  OR “care management”:ti,ab OR “comprehensive care”:ti,ab OR “care planning”:ti,ab OR “disease management”:ti,ab) | 1,021 |
| 15 | Identify diagnosis and diagnostic imaging  subheadings | [mh /DI] AND [mh/DG] | 74,049 |
| 16 | Exclude  diagnostic studies | #14 NOT (#15 OR “diagnos*”:ti,ab OR “diagnostic”:ti,ab) | 835 |
| 17 | Exclude *in vitro* and *in vivo*studies | #16 NOT (“*in vitro*”:ti,ab OR “*in vivo*”:ti,ab) | 834 |
| 18 | Limits | Studies with a Cochrane library publication date from Jan 2014 to Nov 2019, in Cochrane Reviews and Cochrane Trials | 633 |
