# Supplementary material for: Digital Health Interventions to Enhance Prevention in Primary Care: Scoping Review
Source: JMIR Med Inform. 2022 Jan 21;10(1):e33518. doi: 10.2196/33518 (PMC8817213; doi:10.2196/33518)
Supplement: Multimedia Appendix 5 [file medinform_v10i1e33518_app5.docx]

Multimedia Appendix 5. Updated MEDLINE search via PubMed.

| Search no. | Facet | Search Terms | Search Results (July 19, 2020) |
| --- | --- | --- | --- |
| 1 | Primary care | “primary health care”[MeSH] OR "Physicians, Primary Care"[MeSH] OR “primary care”[tiab] | 228,216 |
| 2 | Primary care specialty – family practice | "Family Practice"[MeSH] OR “family practice”[tiab] OR "Physicians, Family"[MeSH] OR “family physician*”[tiab] OR “family medicine”[tiab] OR “general medicine”[MeSH] or “general medicine”[tiab] | 95,763 |
| 3 | Primary care specialty – internal medicine | ("Internal Medicine"[MeSH] OR “internal medicine”[tiab]) AND (“primary care”[tiab] OR “family practice”[tiab] OR “family medicine”[tiab] OR “general medicine”[tiab]) | 5,479 |
| 4 | Primary care specialty – pediatrics | "Pediatrics"[MeSH] OR “pediatric*”[tiab] OR "Pediatricians"[MeSH] OR “pediatrician*”[tiab]  AND (“primary care”[tiab] OR “family practice”[tiab] OR “family medicine”[tiab] OR “general medicine”[tiab]) | 7,670 |
| 5 | Primary care specialty - geriatrics | "Geriatrics"[MeSH] OR “geriatric*”[tiab] OR "Geriatricians"[MeSH] OR “geriatrician*”[tiab]  AND (“primary care”[tiab] OR “family practice”[tiab] OR “family medicine”[tiab] OR “general medicine”[tiab]) | 2,353 |
| 6 | Non-physician primary care professionals | ("Nurse Practitioners"[MeSH] OR “nurse*”[tiab] OR “Physician Assistants"[MeSH] OR “physician assistant*”[tiab] OR "Pharmacists"[MeSH] OR “pharmacist*”[tiab]) AND (“primary care”[tiab] OR “family practice”[tiab] OR “family medicine”[tiab] OR “general medicine”[tiab]) | 13,373 |
| 7 | All primary care professionals | #1 OR #2 OR #3 OR #4 OR #5 OR #6 | 302,337 |
| 8 | Digital health and health behavior technologies synonyms | "digital health"[tiab] OR "digital health intervention"[tiab] OR "digital behavior change"[tiab] OR "digital behaviour change"[tiab] OR "digital health technology"[tiab]) | 1,935 |
| 9 | Specific types of digital health technologies outlined by FDA/WHO | “Electronic Health Records”[MeSH] OR “electronic health record*”[tiab] OR “personal health record*”[tiab] OR “electronic medical record*”[tiab] OR “EMR”[tiab] OR “EHR”[tiab] OR "Health Records, Personal"[MeSH] OR  "Computer Security"[Mesh] OR "data security"[tiab] OR "cybersecurity"[tiab] OR "cyber security"[tiab] OR "data protect*"[tiab] OR "data encrypt*"[tiab] OR  "Cloud Computing"[Mesh] OR "cloud computing"[tiab] OR "cloud process*"[tiab] OR "cognitive comput*"[tiab] OR  "Patient Portals"[Mesh] OR "patient web portal*"[tiab] OR "patient web-portal*"[tiab] OR "patient portal*"[tiab] OR "web portal*"[tiab] OR  "mobile technolog*"[tiab] OR "Telemedicine"[Mesh] OR "telemedicine"[tiab] OR "telehealth*"[tiab] OR "mobile health"[tiab] OR "mHealth"[tiab] OR "eHealth"[tiab] OR "m-Health"[tiab] OR "mobile-health"[tiab] OR "telecommunication*"[tiab] OR ((app OR application*) n3 (smartphone* or smart-phone or mobile* or phone*)) OR  "Decision Support Systems, Clinical"[Mesh] OR "clinical decision support"[tiab] OR "decision support system"[tiab] OR  "Health Information Exchange"[MeSH] OR “health information exchange*”[tiab] OR “electronic health information”[tiab] OR “electronic health communication*”[tiab] OR “health information interoperability”[MeSH] OR “interoperability” OR  “patient monitor*”[tiab] OR “wearables”[tiab] OR “activity monitor*”[tiab] OR “sensor*”[tiab] OR “physiologic monitoring”[MeSH] OR “physiologic monitoring”[tiab] | 482,092 |
| 10 | Specific types of digital health technologies not outlined by the FDA/WHO but are of interest | “Artificial Intelligence”[Mesh] OR “artificial intelligence”[tiab] OR “machine intelligence”[tiab] OR “computational intelligence”[tiab] OR “Machine Learning”[MeSH] OR  “machine learning”[tiab] OR “machine-learning”[tiab] OR “natural language processing”[tiab] OR “neural network*”[tiab] OR “quantified self”[tiab] OR “connected health”[tiab] OR “big data”[tiab] OR “gamification”[tiab] OR “social media”[tiab] OR “health 2.0”[tiab] OR “personalized genomics”[tiab] OR “precision medicine”[tiab] OR “precision medicine”[MeSH] OR “individualized medicine”[tiab] OR “internet of things”[tiab] OR “IoT”[tiab] OR “IOT”[tiab] OR  ((“social program*”[tiab] OR "care manage*"[tiab] OR “coordinate* care”[tiab] OR “health benefit*”[tiab] OR insur*[tiab]) AND “digital”[tiab]) | 191,667 |
| 11 | Combine digital health technology strings | #8 OR #9 OR #10 | 654,377 |
| 12 | Combine digital health technology with primary care strings | #7 AND #11 | 13,943 |
| 13 | Prevention and care management studies using digital health in primary care | #12 AND (“prevention and control”[subheading] OR “prevention”[tiab] OR “preventive”[tiab] OR “mass screening”[MeSH] OR “screening”[tiab] OR “preventive health services”[MeSH] OR “Patient Care Management”[MeSH] OR “care management”[tiab] OR “care management”[tiab] OR “comprehensive care”[tiab] OR “care planning”[tiab] or “disease management”[tiab]) | 10,983 |
| 14 | Diagnostic studies | “diagnosis”[MeSH] OR “diagnos*”[tiab] OR “diagnosis”[subheading] or “diagnostic”[tiab] | 10,319,118 |
| 15 | Exclude diagnostic studies | #13 NOT #14 | 6,537 |
| 16 | Exclude *in vitro* and *in vivo* studies | #15 NOT ("in vitro" OR "in vivo") | 6,534 |
| 17 | Exclude narrative reviews | Review[pt] NOT (Cochrane OR systematic or meta-analy*) | 2,374,915 |
| 18 |  | #16 NOT #17 | 6,186 |
| 19 |  | "case reports"[pt] OR "letter"[pt] OR "editorial"[pt] OR "case series"[pt] | 3,518,431 |
| 20 |  | #18 NOT #19 | 5,867 |
| 21 | Limit to studies published after October 15, 2018 to July 19, 2020 | #20 AND (2018/10:2020/07[edat]) | 865 |
| 22 | Limit to studies with abstracts | #20 Filters: Abstracts | 817 |
| 23 | Limit to English | #20 Filters: Abstracts; English | 801 |
| 24 | Limit to studies in humans | #20 Filters: Abstracts; English; Humans | 561 |
