# Supplementary material for: Digital Health Interventions to Enhance Prevention in Primary Care: Scoping Review
Source: JMIR Med Inform. 2022 Jan 21;10(1):e33518. doi: 10.2196/33518 (PMC8817213; doi:10.2196/33518)
Supplement: Multimedia Appendix 7 [file medinform_v10i1e33518_app7.docx]

Multimedia Appendix 7. Updated Embase search.

| Search no. | Facet | Search Terms | Search Results  (July 19, 2020) |
| --- | --- | --- | --- |
| 1 | Primary care | 'primary health care'/exp OR 'primary health care' OR 'general practitioner'/exp OR 'general practitioner':ab,ti OR 'primary care':ab,ti | 320,203 |
| 2 | Primary care specialty – family practice | 'general practice'/exp OR 'general practice':ab,ti OR 'general practitioner'/exp OR 'family practice':ab,ti OR 'family physician':ab,ti OR 'family medicine'/exp OR 'general medicine':ab,ti | 199,809 |
| 3 | Primary care specialty – internal medicine | ('internal medicine'/exp OR 'internal medicine':ab,ti) AND 'primary care':ab,ti | 9,875 |
| 4 | Primary care specialty – pediatrics | 'pediatrics'/exp OR 'pediatrician'/exp OR pediatric*:ab,ti OR pediatrician*:ab,ti | 521,557 |
| 5 | Primary care specialty - geriatrics | 'geriatrics'/exp OR 'geriatrician'/exp OR 'geriatrics':ab,ti OR 'geriatrician*':ab,ti | 64,866 |
| 6 | Non-physician primary care professionals | ('nurse practitioner'/exp OR 'nurse':ab,ti OR 'physician assistant'/exp OR 'physician assistant':ab,ti OR 'pharmacist'/exp OR 'pharmacist':ab,ti) AND ('primary care':ab,ti OR 'family practice':ab,ti OR 'family medicine':ab,ti OR 'general medicine':ab,ti OR 'general practice':ab,ti) | 15,155 |
| 7 | All primary care providers | #1 OR #2 OR #3 OR #4 OR #5 OR #6 | 961,896 |
| 8 | Digital health and health behavior technologies synonyms | ‘digital health’:ab,ti OR ‘digital health intervention’:ab,ti OR ‘digital behavior change’:ab,ti OR ‘digital behaviour change’:ab,ti OR ‘digital health technology’:ab,ti | 1,672 |
| 9 | Specific types of digital health technologies outlined by FDA/WHO | 'medical record'/exp OR 'electronic health record*':ab,ti OR 'personal health record*':ab,ti OR 'electronic medical record':ab,ti OR 'emr':ab,ti OR 'ehr':ab,ti OR  'computer security'/exp OR 'data security':ab,ti OR 'cybersecurity':ab,ti OR 'cyber security':ab,ti OR 'data protect*':ab,ti OR 'data encrypt*':ab,ti OR  'cloud computing'/exp OR 'cloud computing':ab,ti OR 'cloud process*':ab,ti OR 'cognitive comput*':ab,ti OR  'patient web portal*':ab,ti OR 'patient web-portal*':ab,ti OR 'patient portal*':ab,ti OR 'web portal*':ab,ti OR  'mobile technolog*':ab,ti OR 'telehealth’/exp OR ‘telemedicine':ab,ti OR 'telehealth*':ab,ti OR 'mobile health':ab,ti OR 'mHealth':ab,ti OR 'ehealth':ab,ti OR 'm-health':ab,ti OR 'mobile-health':ab,ti OR ' mobile application':ab,ti OR  'decision support system’/exp OR 'clinical decision support':ab,ti OR 'decision support system*':ab,ti OR  'medical information system'/exp OR 'health information exchange*':ab,ti OR 'electronic health information':ab,ti OR 'electronic health communication*':ab,ti OR ‘data interoperability'/exp OR 'interoperability':ab,ti OR  'patient monitor*':ab,ti OR 'wearables':ab,ti OR 'activity monitor*':ab,ti OR (‘health’:ab,ti AND 'sensor*':ab,ti) OR 'physiologic monitoring'/exp OR 'physiologic monitoring':ab,ti | 400,546 |
| 10 | Specific types of digital health technologies not outlined by the FDA/WHO but are of interest | 'artificial intelligence'/exp OR 'artificial intelligence':ab,ti OR 'machine intelligence':ab,ti OR 'computational intelligence':ab,ti OR 'machine learning'/exp OR 'machine learning':ab,ti OR 'machine-learning':ab,ti OR 'natural language processing':ab,ti OR 'neural network*':ab,ti OR 'quantified self':ab,ti OR 'connected health':ab,ti OR 'big data':ab,ti OR 'gamification':ab,ti OR 'social media':ab,ti OR 'health 2.0':ab,ti OR 'personalized genomics':ab,ti OR 'personalized medicine'/exp OR 'precision medicine':ab,ti OR 'individualized medicine':ab,ti OR 'internet of things':ab,ti OR 'iot':ab,ti OR (('social program*:ab,ti or care manage*':ab,ti OR 'coordinate* care':ab,ti OR 'health benefit*':ab,ti OR 'insur*':ab,ti) AND 'digital':ab,ti) | 317,203 |
| 11 | Combine digital health technology strings | #8 OR #9 OR #10 | 700,496 |
| 12 | Combine digital health technology and primary care | #7 AND #11 | 51,315 |
| 13 | Prevention and control to include screening and diagnosis using digital health in primary care | #12 AND (‘prevention’/exp OR ‘prevention‘:ab,ti OR ‘preventive‘:ab,ti OR ‘mass screening’/exp OR ‘screening‘:ab,ti OR ‘preventive health service’/exp OR ‘patient care planning’/exp OR ‘care management’:ab,ti OR ‘comprehensive care’:ab,ti OR ‘care planning’:ab,ti OR ‘disease management’:ab,ti) | 11,034 |
| 14 | Diagnostic studies | 'diagnosis'/exp OR 'diagnos*':ab,ti OR 'diagnostic':ab,ti | 8,423,348 |
| 15 | Exclude diagnostic studies | #13 NOT #14 | 6,281 |
| 16 | Exclude in vitro and in vivo studies | #15 NOT (‘in vitro’:ab,ti OR ‘in vivo’:ab,ti) | 6,269 |
| 17 | Exclude narrative reviews | [review]/lim NOT (Cochrane OR systematic or meta-analy*) | 2,506,202 |
| 18 |  | #16 NOT #17 | 5,852 |
| 19 | Exclude case reports, case series, letters, and editorials | ‘case report’/exp OR ‘case report*’ OR ‘case study’/exp OR ‘case series’ | 2,758,426 |
| 20 |  | #18 NOT #19 | 5,724 |
| 21 | Limit to articles with abstract | #20 AND [abstracts]/lim | 5,297 |
| 22 | Limit to update search since 10/15/2018 to 8/1/2020 | #21 AND [10-15-2018]/sd NOT [8-1-2020]/sd | 1,315 |
| 23 | Limit to humans | #22 AND [humans]/lim | 1,298 |
| 24 | Limit to English | #23 AND [English]/lim | 1,287 |
| 25 | Eliminate duplicates from Medline via Pubmed | #24 AND [embase]/lim | 1,107 |
| 26 | Eliminate conference proceedings, editorials, and letters | #25 NOT ([conference abstract]/lim OR [conference paper]/lim OR [conference review]/lim OR [editorial]/lim OR [erratum]/lim OR [letter]/lim OR [note]/lim) | 477 |
