# Supplementary material for: Digital Health Interventions to Enhance Prevention in Primary Care: Scoping Review
Source: JMIR Med Inform. 2022 Jan 21;10(1):e33518. doi: 10.2196/33518 (PMC8817213; doi:10.2196/33518)
Supplement: Multimedia Appendix 8 [file medinform_v10i1e33518_app8.docx]

Multimedia Appendix 8. Inclusion/exclusion criteria.

|  | **Inclusion** | **Exclusion** |
| --- | --- | --- |
| Population | The study examines patient/consumers, providers, and/or population stakeholders in primary care. | The study does not describe a population, that receives, treats, or manages patients receiving primary care. |
| Intervention | **The study uses a minimum of one FDA/WHO or non-regulated digital health technology facet.**  The study utilizes the following FDA/WHO recognized digital health components for prevention:   - telehealth (e-Health, telemonitoring, telecare, telemedicine); telemedicine with communication via interactive voice response (IVR) will be allowed. - mHealth - health information technology (HIT): - storage - cybersecurity - EHR/EMR/HIE - software as a medical device: - clinical decision support tools - mHealth apps - wireless medical devices - medical device interoperability - medical device data systems - medical imaging   The study does not have to be limited to FDA/WHO recognized digital components, it can include other digital health facets that examine prevention such as:   - data analytics - predictive - prescriptive - artificial intelligence - weak - strong - Digital behavior change interventions (DBCIs) (e.g., non-medical device status for eHealth, health/wellness apps; quantified self) - big data - gamification - non-regulated sensors and wearables - Health 2.0 / social media - personalized genomics - precision medicine - quantified self - Studies related to care management will be included if they serve primary, secondary, tertiary, or quaternary prevention outcomes. Medical imaging will be allowed if it was intended for prevention (e.g., screening and/or surveillance). | Studies with digital health interventions regarding treatment and diagnosis.  Notable exception for diagnosis, if it is screening: Studies with preventive care interventions regarding screening will be included.  Medical imaging for diagnosis and telehealth studies only using non-cellular telephone communication. |
| Comparison | No comparisons are required. | N/A |
| Outcomes | **The study reports on health, healthcare performance, and/or DHI implementation outcomes.**  **Health** domain areas at individual or population-level:   - emotional - environmental - intellectual - physical - social - spiritual   **Healthcare performance** areas of quality, efficiency, access, or utilization to identify the following measures:   - structural - process - outcomes   **DHI implementation** will include:   - acceptability - adoption - appropriateness - costs - feasibility - fidelity - penetration - sustainability | The study does not report on outcomes related to health domains, healthcare performance measures, or DHI implementation. |
| Situation | No study limits on geography, global findings will be included. | None. |
| Limits – study design | The publication describes a primary study or systematic review (with the same inclusion criteria) with or without meta-analyses. | The publication describes a study design other than a primary study or systematic review. Case studies, case series, narrative reviews, editorials, or policy pieces. |
| Limits - language | The publication is in English. | The publication is in a language other than English. |
| Limits - human | The study is conducted in humans. | The study is not conducted in humans or examines human *in vitro* cells. |
| Limits – time; published in the last six years | The study was published in the last six years (January 1, 2014 and July 31, 2020) | The publication date is before January 1, 2014 or after August 2020. |
