# Supplementary material for: Digital Health Interventions to Enhance Prevention in Primary Care: Scoping Review
Source: JMIR Med Inform. 2022 Jan 21;10(1):e33518. doi: 10.2196/33518 (PMC8817213; doi:10.2196/33518)
Supplement: Multimedia Appendix 9 [file medinform_v10i1e33518_app9.docx]

**Multimedia Appendix 9.** Description of digital health intervention categories.

| **Digital Health Technology Facet** | **Description(s)** |
| --- | --- |
| Telehealth / connected health / eHealth | Telehealth / connected health (includes telemedicine, telemonitoring, and telecare) refers to remote clinical services, such as electronic consultation, transmission of medical data for diagnosis and management, and remote patient monitoring. Application of IVR to telehealth.  eHealth refers to the use of information and communication technologies such as “internet, wireless networks, cell phones, and other communication media” for health and health-related fields (World Health Organization, 2016). |
| mHealth (includes health and wellness apps)  (mobile apps may or may not be a *medical device*) | “The use of mobile wireless technologies for public health,” such as SMS, GPS, and smartphone applications (apps) (World Health Organization, 2018). |
| Health IT | The application of information processing and transfer in health care, including EMR/EHR, patient portals, and field-specific information systems such as radiology information systems. This includes storage (e.g., cloud-based, data warehouses) and cybersecurity. |
| EHR/EMR/HIE | A subset of Health IT, electronic health records (EHR) or electronic medical records (EMR) serve to systematically store and transmit patient information, including test results, demographics, medical history, and billing information.  The health exchange of electronic records between providers and health systems is facilitated by health information exchange (HIE), which is a prerequisite for interoperability. |
| Software as a medical device (ie, clinical decision support, mHealth apps) | The Food and Drug Administration (FDA) has long regulated software that meets the definition of a device in section 201(h) of the Federal Food, Drug, and Cosmetic Act (FD&C Act), including software that is intended to provide decision support for the diagnosis, treatment, prevention, cure, or mitigation of diseases or other conditions (often referred to as clinical decision support software).  FDA recognizes that the term "clinical decision support" or "CDS" is used broadly and in different ways, depending on the context. CDS provides health care professionals (HCPs) and patients with knowledge and person-specific information, intelligently filtered or presented at appropriate times, to enhance health and health care.[1](https://www.fda.gov/regulatory-information/search-fda-guidance-documents/clinical-decision-support-software#ft1) In the Food and Drug Administration Safety and Innovation Act (FDASIA) Health IT Report of 2014, CDS is described as a variety of tools including, but not limited to:   - computerized alerts and reminders for providers and patients; - clinical guidelines; - condition-specific order sets; - focused patient data reports and summaries; - documentation templates; - diagnostic support; and - contextually relevant reference information.   A mobile app is considered a medical device if its intended use is for “the diagnosis of disease or other conditions, or the cure, mitigation, treatment, or prevention of disease, or is intended to affect the structure or any function of the body of man” (U.S. Food and Drug Administration, 2015). These fall under FDA guidance, and examples include:   - health records access, - educational tools, and - emergency response systems   Mobile apps that do not fall under the definition of medical devices but can assist users “to log, record, track, evaluate, or make decisions or behavioral suggestions related to developing or maintaining general fitness, health or wellness,” such as diet and nutrition coaching or menstrual cycle tracking (U.S. Food and Drug Administration, 2015). |
| Wireless medical devices | Radio frequency (RF) wireless medical devices (ie, sensors/wearables) perform at least one function that utilizes wireless RF communication to support health care delivery.  Examples of functions that can utilize wireless technology include controlling and programming a medical device, monitoring patients remotely, or transferring patient data from the medical device to another platform such as a cell phone.  Wireless devices can be implanted to control heart rhythms, monitor hypertension, provide functional electrical stimulation of nerves, operate as sensors for glucose, monitor bladder and cranial pressures.  External devices can monitor vital signs, assist movement of artificial limbs, and function as miniature base stations for the collection and transmission of various physiological parameters. Miniature transponders can be embedded in pills of monitoring.  Short Range devices for patient monitoring, control, and diagnostics:   - Inductive Implants: Historically, inductive implantable medical devices have been used to control or monitor cardio activity. Most devices operate in the bands below 200 kHz and communicate at distances of less than one foot from the patient’s body. - Medical Device Radiocommunication Service (formerly “MICS”): Allocated in 1999 for licensed communication between body implants and a nearby controller, the FCC added more frequencies to this service in 2009 for use by body-worn monitoring devices. These devices operate in the 401-406 MHz band at distances up to about 10 feet. - Wi-Fi, Bluetooth, and Zigbee: These unlicensed technologies are commonly used with cell phones, handheld devices and personal computers, but can also be used for implanted or body-worn medical devices. These devices operate in the 902-928, 2400-2483.5 and 5725- 5850 MHz bands at distances up to a few hundred feet. - Ultra-Wideband: New uses of unlicensed ultra-wideband technologies are starting to emerge for medical telemetry and imaging applications. These devices operate at very low power in almost any region of the spectrum at distances up to a few feet. - Medical Micropower Networks: In November 2011 the FCC allocated new spectrum to accommodate the operation of implanted microstimulator devices that might lead to the creation of an artificial nervous system that could restore mobility to paralyzed limbs. These devices operate in the 413-457 MHz band at distances up to a few feet. - Medical Body Area Networks: In May 2012 the FCC allocated new spectrum to allow a wireless personal area network (“PAN”) of multiple body sensors to monitor or control patient functions. These devices operate in the 2360-2400 MHz band at distances up to a few feet.   Long-range medical telemetry:   - Wireless Medical Telemetry (WMTS): WMTS uses unlicensed spectrum to communicate data from body sensors to remote monitoring locations. These devices operate in various bands between 600 and 1432 MHz band at distances up to several hundred feet. - Worldwide Interoperability for Internet Access (WiMAX): Often referred to as a “last mile” broadband access technology, WiMAX provides wireless transmission using a variety of transmission modes, from point-to-multipoint links to portable and fully mobile Internet access. The technology provides up to 70 Mbps broadband at distances over several kilometers. The technology is based on the IEEE 802.16 standard (also called Broadband Wireless Access) and uses frequencies around 2.5 GHz in the U.S. |
| Medical device interoperability | The ability to securely share health information and data within and across organizational boundaries, including that between physicians, care facilities, labs, pharmacy, and patients (Healthcare Information and Management Systems Society definition).  As per FDA, medical device interoperability is the ability to safely, securely, and effectively exchange and use information among one or more devices, products, technologies, or systems. This exchanged information can be used in a variety of ways including display, store, interpret, analyze, and automatically act on or control another product. |
| Medical device data systems | Medical Device Data Systems (MDDS) are hardware or software products intended to transfer, store, convert formats, and display medical device data. A MDDS does not modify the data or modify the display of the data, and it does not by itself control the functions or parameters of any other medical device. MDDS may or may not be intended for active patient monitoring.  Per section 520(o)(1)(D) of the Federal Food, Drug, and Cosmetic Act:   - Software functions that are *solely intended* to transfer, store, convert formats, and display medical device data or medical imaging data, are not devices and are not subject to FDA regulatory requirements applicable to devices. The FDA describes these software functions as "Non-Device-MDDS." - Hardware functions that are *solely intended* to transfer, store, convert formats, and display medical device data or results are "Device-MDDS."   Examples of Non-Device-MDDS include software functions that:   - Store patient data, such as blood pressure readings, for review at a later time; - Convert digital data generated by a pulse oximeter into a format that can be printed; and - Display a previously stored electrocardiogram for a particular patient. |
| Medical imaging | Imaging techniques for diagnosis and treatment purposes, such as MRI, ultrasound, and endoscopy. |
