# Supplementary figures and images for: Digital Health Interventions to Enhance Prevention in Primary Care: Scoping Review
Source: JMIR Med Inform. 2022 Jan 21;10(1):e33518. doi: 10.2196/33518 (PMC8817213; doi:10.2196/33518)

**Multimedia Appendix 10.** Data extraction forms.


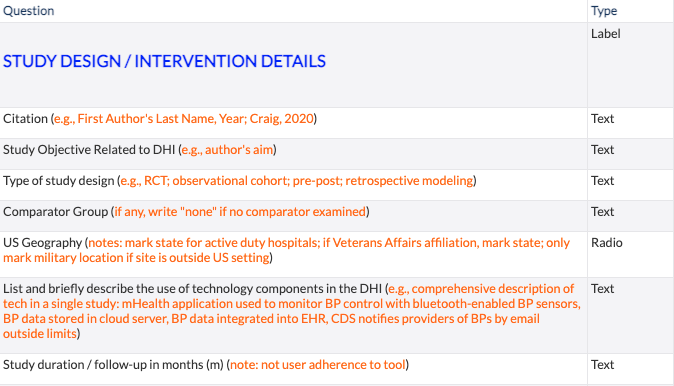


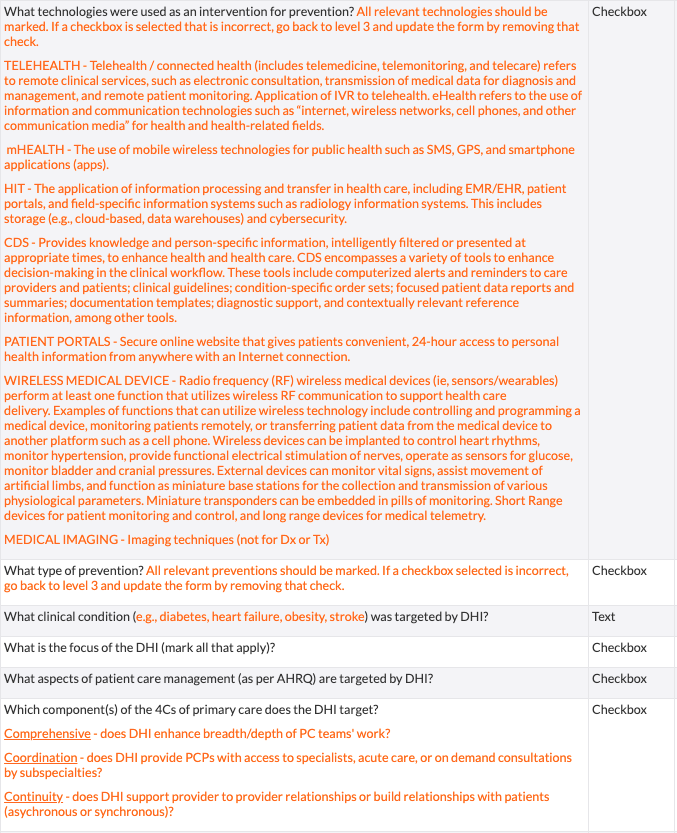


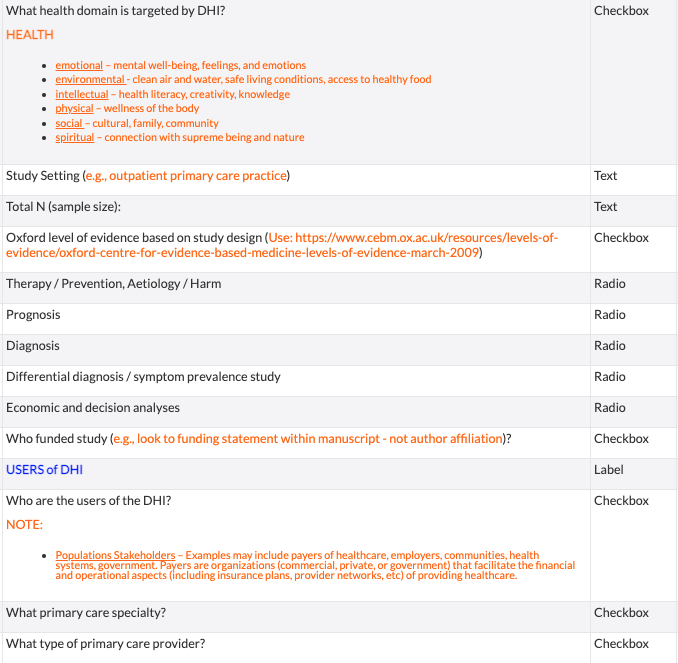


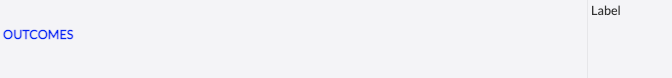


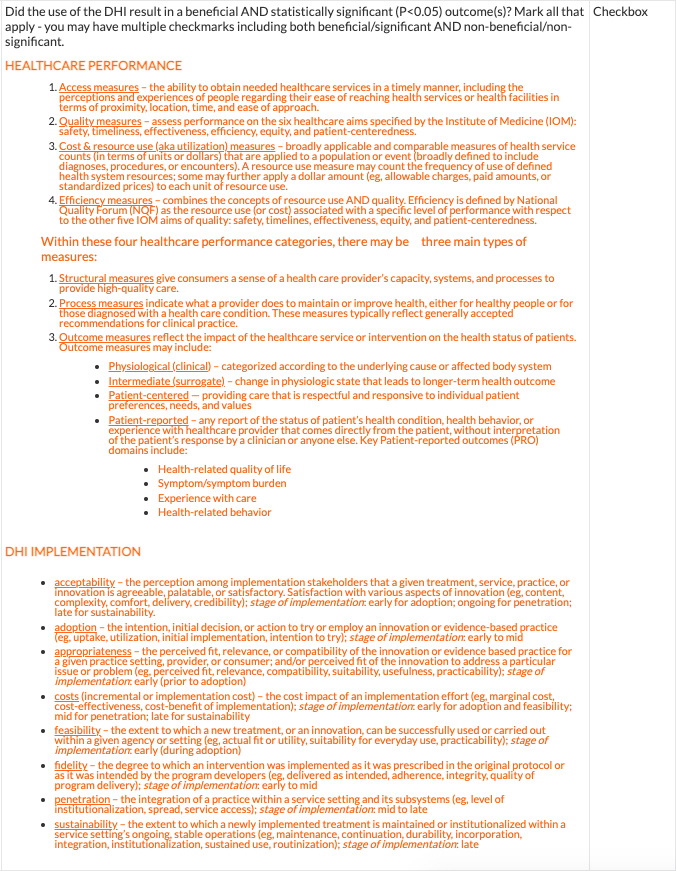


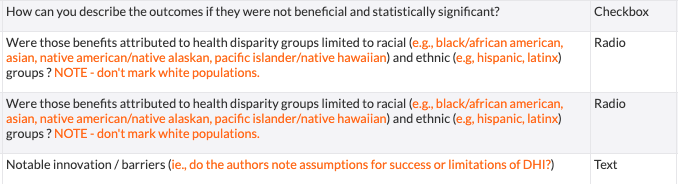


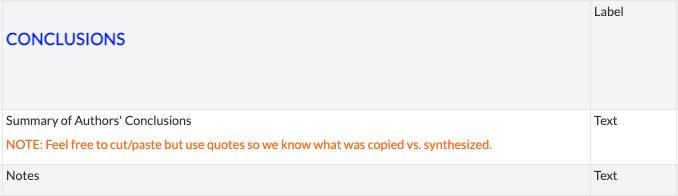

Supplement: Multimedia Appendix 10 [file medinform_v10i1e33518_app10.docx]
