# Supplementary material for: Digital Health Interventions to Enhance Prevention in Primary Care: Scoping Review
Source: JMIR Med Inform. 2022 Jan 21;10(1):e33518. doi: 10.2196/33518 (PMC8817213; doi:10.2196/33518)
Supplement: Multimedia Appendix 11 [file medinform_v10i1e33518_app11.docx]

**Multimedia Appendix 11.** Abstracted results from included articles.

| First author, year | DHI type | Prevention | 4Cs ^a^ | Healthcare outcomes^b^ | Implementation outcomes^b^ |
| --- | --- | --- | --- | --- | --- |
| Adams, 2014 [17] | Telehealth, HIT, CDS | 2°, 3°, 4° | Comprehensive | ↑* | ↑* |
| Afshar, 2019 [18] | Telehealth, other | 2° | Comprehensive | - | - |
| Ahern, 2016 [19] | mHealth | 2° | Comprehensive |  | ↑* |
| Aikens, 2015 [20] | Telehealth | 3° | Comprehensive Continuous | ↑* | - |
| Akenroye, 2017 [21] | HIT, CDS | 2° | Comprehensive | - | - |
| Albu, 2017 [22] | HIT, CDS | 2° | Comprehensive | - | ↑* |
| Aleem, 2015 [23] | HIT | 2° | Comprehensive Coordinated | ↑* | - |
| Allen, 2017 [24] | Telehealth, HIT, other | 3° | Comprehensive Continuous Coordinated | - | ↑* |
| Anderson, 2018 [25] | Telehealth, HIT | 3° | Coordinated | ↑* | ↑* |
| Apter, 2019 [26] | HIT, patient portals | 3° | First Contact Continuous | ↑* | - |
| Bachhuber, 2017 [27] | HIT, CDS | 2°, 3° | Comprehensive Continuous | - | ↑* |
| Bae, 2017 [29] | HIT, CDS | 1° | Comprehensive | ↑* | ↑* |
| Bae, 2018 [28] | HIT, CDS | 3° | Comprehensive | ↑* | ↑* |
| Baker, 2019 [30] | Telehealth | 3° | First Contact Comprehensive | ↑* | - |
| Bar-Shain, 2015 [31] | mHealth, HIT | 1° | Comprehensive Coordinated | - | ↑* |
| Barclay, 2019 [32] | HIT | 2° | Continuous | - | ↑* |
| Barton, 2018 [33] | Telehealth, HIT | 3° | Comprehensive Coordinated | ↑* | - |
| Beasley, 2019 [34] | Telehealth, other | 3° | First Contact Comprehensive Continuous | - | - |
| Bennett, 2018 [35] | Telehealth, mHealth, HIT, WMD | 3° | Comprehensive Continuous | ↑* | - |
| Benson 2018 [36] | Telehealth, HIT | 2° | Comprehensive | ↑* | - |
| Berkley, 2017 [37] | HIT, CDS | 2° | Comprehensive | - | - |
| Bhat, 2018 [38] | HIT, other | 3° | Comprehensive Continuous Coordinated | - | - |
| Bondurant, 2017 [39] | Telehealth, mHealth, HIT | 1° | Comprehensive | ↑* | ↑* |
| Bose-Brill, 2016 [41] | HIT, CDS | 4° | Coordinated | - | ↑* |
| Bose-Brill, 2018 [40] | HIT, patient portals | 4° | Comprehensive | ↑* | ↑* |
| Bouskill, 2018 [42] | Telehealth | 2° | Comprehensive Coordinated | - | - |
| Brantley, 2019 [43] | Telehealth | 3° | Continuous Coordinated | ↑* | ↑* |
| Bratic, 2019 [44] | HIT, CDS | 1° | Comprehensive | ↑* | - |
| Brayboy, 2017 [45] | mHealth | 1°, 2°, 3° | Comprehensive | - | ↑* |
| Breitenstein, 2019 [46] | mHealth | 1° | Comprehensive | - | - |
| Brown, 2015 [47] | HIT, other | 1° | First Contact Coordinated | - | ↑* |
| Brunette, 2015 [48] | CDS, other | 3° | Comprehensive Continuous | - | ↑* |
| Buist, 2014 [49] | Telehealth, HIT, patient portals, CDS | 3° | Comprehensive Continuous | - | - |
| Bulbin, 2018 [50] | HIT, CDS | 3° | Comprehensive Continuous Coordinated | ↑* | ↑* |
| Burdick, 2017 [51] | HIT, CDS | 2°, 3° | Comprehensive Coordinated | - | ↑* |
| Carswell, 2019 [52] | mHealth, other | 2° | Comprehensive | - | - |
| Cawthon, 2014 [53] | Telehealth, HIT | 2°, 4° | Comprehensive | - | - |
| Chambers, 2015 [54] | HIT | 2°, 3° | First Contact Comprehensive | - | ↑* |
| Chouteau, 2019 [55] | HIT | 3° | Continuous | - | - |
| Chung, 2019 [56] | HIT | 2° | Comprehensive | ↑* | ↑* |
| Church, 2018 [57] | HIT, other | 1° | Comprehensive | ↑* |  |
| Coker 2019 [58] | Telehealth, mHealth | 2°, 3° | First Contact Coordinated | ↑* | - |
| Collins, 2018 [59] | HIT, CDS | 2°, 3° | Comprehensive Continuous | ↑* | - |
| Condren, 2014 [60] | HIT | 4° | Coordinated | ↑* | ↑* |
| Conklin, 2020 [61] | HIT, CDS | 2° | Comprehensive | - | - |
| Cordova 2018 [62] | mHealth, other | 1° | Comprehensive | - | ↑* |
| Curtis, 2016 [63] | HIT | 3° | Comprehensive | - | ↑* |
| Dahne, 2019 [64] | mHealth, other | 3° | Comprehensive | ↑* | - |
| Dean, 2016 [65] | Telehealth, CDS | 3°, 4° | Coordinated | - | - |
| DeCamp, 2020 [66] | mHealth | 1° | First Contact Comprehensive Coordinated | ↑* | - |
| De Santes, 2017 [67] | HIT, CDS | 2° | Comprehensive | ↑* | - |
| Devries, 2017 [68] | HIT | 4° | Comprehensive Coordinated | ↑* | ↑* |
| Dexter, 2019 [69] | Telehealth | 3° | First Contact Comprehensive | ↑* | - |
| Diaz, 2019 [70] | CDS | 2° | Comprehensive | ↑* | - |
| Dischinger, 2015 [71] | HIT, CDS | 2° | Comprehensive | ↑* | - |
| Dixon, 2014 [72] | HIT, CDS | 3° | Comprehensive | - | - |
| Dombkowski, 2017 [73] | Telehealth, HIT | 1° | Comprehensive | ↑* | - |
| Donovan, 2016 [74] | HIT, CDS | 4° | Continuous Coordinated | - | - |
| Duquaine, 2015 [75] | HIT, CDS | 3° | Comprehensive Coordinated | - | - |
| Dwinnells, 2017 [76] | mHealth | 2° | Comprehensive | - | ↑* |
| Eckman, 2016 [77] | Telehealth, HIT, CDS | 3° | Comprehensive | - | - |
| Eckman, 2016 [78] | HIT, CDS, other | 3°, 4° | Comprehensive | ↑* | - |
| Emerson, 2015 [79] | mHealth, HIT, WMD | 3° | Comprehensive Continuous Coordinated | - | - |
| Engel, 2015 [80] | Telehealth, HIT, WMD | 3° | Comprehensive Continuous | - | ↑* |
| Fanizza, 2018 [81] | HIT | 3° | Continuous Coordinated | ↑* | ↑* |
| Federman, 2017 [83] | HIT, CDS | 2° | Comprehensive | ↑* | - |
| Federman, 2018 [82] | Telehealth, HIT, patient portals | 1°, 3°, 4° | Comprehensive Continuous Coordinated | - | ↑* |
| Fiks, 2016 [85] | HIT | 1° | Comprehensive | - | ↑* |
| Fiks, 2016 [84] | Patient portals, CDS | 3° | Comprehensive Coordinated | - | - |
| Fiore, 2019 [86] | HIT | 2°, 3° | Comprehensive | - | ↑* |
| Fitzpatrick, 2017 [87] | HIT, CDS | 3° | Comprehensive Coordinated | - | - |
| Flocke, 2019 [88] | Telehealth, HIT, CDS, other | 3° | Comprehensive Coordinated | ↑* | - |
| Flynn, 2017 [89] | Telehealth | 3° | Comprehensive Continuous Coordinated | - | - |
| Frank, 2015 [90] | Telehealth | 3° | Comprehensive Continuous | - | ↑* |
| Fried, 2017 [91] | HIT, CDS | 4° | Comprehensive Coordinated | ↑* | ↑* |
| Ganz, 2015 [92] | HIT, CDS | 2° | Comprehensive | - | - |
| Geboy, 2019 [93] | CDS | 2° | Comprehensive Coordinated | - | ↑* |
| Gill, 2019 [94] | HIT, CDS | 3° | Comprehensive | ↑* | - |
| Gold, 2014 [95] | HIT, CDS | 3° | Comprehensive Continuous | - | ↑* |
| Goleman, 2018 [96] | HIT, CDS | 1° | Comprehensive | ↑* | ↑* |
| Grant, 2015 [97] | HIT, other | 3° | Comprehensive Continuous | ↑* | - |
| Greene, 2019 [98] | mHealth, WMD, other | 2°, 3° | Comprehensive | - | ↑* |
| Gustafson, 2014 [99] | mHealth | 3° | Comprehensive | ↑* | - |
| Ha, 2018 [100] | HIT, CDS | 2° | Comprehensive | ↑* | - |
| Haas, 2015 [102] | HIT, other | 3° | Comprehensive Continuous Coordinated | - | ↑* |
| Haas, 2017 [101] | Telehealth, HIT | 1°, 2° | Comprehensive | ↑* | - |
| Halterman, 2018 [103] | Telehealth | 3° | First Contact Comprehensive Continuous | ↑* | ↑* |
| Hark, 2017 [105] | Telehealth, HIT | 2° | Comprehensive Continuous | - | ↑* |
| Hark, 2018 [104] | Telehealth | 2° | First Contact Comprehensive | - | ↑* |
| Hark, 2019 [106] | Telehealth | 2° | First Contact Comprehensive | ↑* | - |
| Hatef, 2018 [107] | Telehealth | 2° | First Contact Comprehensive Coordinated | ↑* | - |
| Hay, 2018 [108] | CDS, other | 3° | Comprehensive Coordinated | ↑* | ↑* |
| Hayek, 2014 [109] | HIT | 4° | Continuous | ↑* | ↑* |
| Hess, 2014 [110] | HIT, patient portals, CDS | 2°, 3° | Comprehensive Continuous | - | - |
| Heyworth, 2014 [111] | Telehealth | 2° | Comprehensive | ↑* | - |
| Hojat, 2020 [112] | Telehealth, HIT, patient portals, CDS | 2° | Comprehensive | ↑* | - |
| Honaker, 2018 [113] | mHealth, HIT, CDS | 2° | Comprehensive | ↑* | ↑* |
| Honaker, 2019 [114] | HIT, CDS | 2° | Comprehensive Coordinated | - | - |
| Hoopes, 2018 [115] | HIT, other | 3° | Comprehensive Continuous Coordinated | - | ↑* |
| Jain 2019 [116] | HIT | 2° | Comprehensive | - | - |
| Jani, 2017 [117] | Telehealth, HIT | 2° | Comprehensive Continuous | - | ↑* |
| Jenssen, 2016 [118] | HIT, CDS | 2°, 3° | Comprehensive | ↑* | ↑* |
| Jenssen, 2019 [119] | HIT | 3° | Comprehensive | - | ↑* |
| Jetelina, 2018 [120] | HIT, CDS | 2°, 3° | Comprehensive Coordinated | ↑* | ↑* |
| Jones, 2018 [121] | HIT, CDS | 2° | Comprehensive | - | ↑* |
| Kamo, 2017 [122] | HIT | 4° | Comprehensive Coordinated | ↑* | ↑* |
| Kapoor, 2018 [123] | Telehealth, HIT, CDS | 3° | Comprehensive | - | - |
| Karas, 2018 [124] | HIT, CDS | 2° | Comprehensive | ↑* | - |
| Kavanagh, 2014 [125] | HIT, other | 1°, 2°, 3° | Comprehensive | - | - |
| Keck, 2020 [126] | Telehealth, HIT | 3° | Comprehensive | - | - |
| Kershaw, 2018 [127] | HIT, CDS | 2° | Comprehensive | ↑* | - |
| Kim, 2018 [128] | HIT, CDS | 1° | Comprehensive | ↑* | - |
| Kinahan, 2017 [129] | HIT | 3° | First Contact Coordinated | ↑* | ↑* |
| Knierim, 2019 [130] | HIT | 3° | Continuous | - | ↑* |
| Krishnan, 2019 [131] | Telehealth | 1°, 2° | Comprehensive | ↑* | ↑* |
| Kroenke, 2019 [132] | HIT, other | 3° | Comprehensive | - | ↑* |
| Kruger, 2017 [133] | HIT | 2° | Comprehensive | - | ↑* |
| Kukafka, 2015 [135] | CDS | 2° | Continuous | - | ↑* |
| Kukafka, 2018 [134] | Telehealth, HIT, CDS | 2°, 3° | Comprehensive Continuous Coordinated | ↑* | - |
| Ladapo, 2015 [136] | other | 2°, 3° | Comprehensive | ↑* | - |
| Landis, 2014 [137] | HIT | 1°, 2° | Comprehensive | - | ↑* |
| Langford, 2019 [138] | mHealth, patient portals | 3° | Comprehensive Continuous Coordinated | - | ↑* |
| Lanpher, 2016 [139] | Telehealth | 3° | Comprehensive | - | - |
| Leddy, 2019 [140] | mHealth | 3° | Continuous | - | ↑* |
| Lepore, 2018 [141] | HIT, CDS | 2°, 3° | Comprehensive | ↑* | - |
| Levy, 2019 [258] | mHealth, HIT, CDS | 2° | Comprehensive | - | - |
| Lewis, 2018 [143] | Telehealth, HIT, CDS | 2° | Comprehensive Continuous | - | ↑* |
| Liebschutz, 2017 [144] | HIT, CDS | 1°, 4° | Comprehensive | ↑* | - |
| Lin, 2016 [145] | Telehealth, HIT, CDS | 1° | Comprehensive | ↑* | - |
| Litke, 2018 [146] | Telehealth, HIT, other | 3° | Comprehensive Continuous Coordinated | ↑* | - |
| Liu, 2019 [147] | Telehealth, imaging | 2° | Comprehensive Coordinated | - | - |
| Looman, 2015 [148] | Telehealth | 3°, 4° | Comprehensive Continuous Coordinated | ↑* | ↑* |
| Lv, 2017 [149] | Telehealth, mHealth, HIT, patient portals, WMD | 3° | Comprehensive Continuous | ↑* | - |
| MacLean, 2018 [150] | HIT, CDS | 2° | Comprehensive | ↑* | - |
| Maddali, 2019 [151] | Telehealth, CDS, other | 3° | Comprehensive | - | - |
| Mahabee-Gittens, 2018 [152] | HIT, CDS | 2°, 3° | Comprehensive | - | ↑* |
| Mahoney, 2018 [153] | mHealth | 3° | Comprehensive Continuous | - | ↑* |
| Mainous, 2018 [154] | HIT, CDS | 2°, 3° | Comprehensive | ↑* | - |
| Mann, 2016 [155] | HIT, CDS | 3° | Comprehensive Continuous | - | ↑* |
| Mansberger, 2015 [156] | Telehealth, HIT, imaging | 2° | Comprehensive Coordinated | ↑* | - |
| Marcelin, 2016 [157] | HIT, CDS | 2° | Comprehensive | ↑* | - |
| Maxwell, 2016 [158] | Telehealth | 3° | Comprehensive Continuous | - | ↑* |
| McAdam-Marx, 2019 [159] | HIT, CDS | 1° | Comprehensive | ↑* | - |
| McGrath, 2016 [160] | HIT | 2° | Comprehensive | - | ↑* |
| McLendon, 2019 [161] | Telehealth, HIT | 3° | First Contact Comprehensive Coordinated | ↑* | - |
| Meeker, 2016 [162] | Telehealth, HIT, CDS | 4° | Comprehensive | ↑* | - |
| Mehta, 2018 [163] | HIT, patient portals | 2° | Comprehensive Continuous | - | - |
| Mera, 2016 [164] | Telehealth, HIT, CDS | 2° | First Contact Comprehensive Coordinated | - | - |
| Militello, 2017 [165] | HIT, CDS | 2° | Comprehensive | - | ↑* |
| Mitchell, 2018 [166] | Telehealth, CDS | 4° | Comprehensive | ↑* | ↑* |
| Molleda, 2017 [167] | Telehealth, HIT | 1° | Comprehensive |  | ↑* |
| Mundt, 2018 [168] | HIT | 3° | Continuous | ↑* | - |
| Mussman, 2015 [169] | HIT | 3° | Coordinated | - | - |
| Mussulman, 2014 [170] | Telehealth | 3° | Comprehensive | - | ↑* |
| Nagykaldi, 2014 [172] | HIT, CDS, other | 1°, 2°, 3° | Comprehensive Coordinated | ↑* | - |
| Nagykaldi, 2017 [171] | HIT | 1°, 2°, 3° | Comprehensive Continuous | - | ↑* |
| Nitsche, 2018 [173] | HIT, CDS | 2° | Comprehensive | - | - |
| Nowalk, 2016 [174] | Telehealth, HIT, CDS | 1° | Comprehensive | - | ↑* |
| O'Connor, 2014 [175] | Telehealth, HIT | 3° | Comprehensive | - | - |
| Ofili, 2018 [176] | Telehealth, mHealth, HIT, other | 3° | Comprehensive Continuous | ↑* | - |
| Okah 2018 [177] | HIT | 2° | Comprehensive | - | - |
| Osofsky, 2017 [178] | Telehealth | 3° | Comprehensive Continuous | - | ↑* |
| Ozanne, 2014 [179] | Telehealth, CDS | 2° | Comprehensive | - | - |
| Peralta, 2020 [180] | HIT | 2° | Comprehensive Coordinated | ↑* | - |
| Perry, 2018 [181] | Telehealth | 3° | Comprehensive Continuous | - | - |
| Peters, 2018 [182] | HIT | 3° | First Contact Coordinated | - | ↑* |
| Petersen, 2017 [183] | HIT | 3° | Comprehensive | ↑* | - |
| Phillips, 2015 [184] | CDS | 3° | Comprehensive Continuous Coordinated | - | ↑* |
| Quanbeck 2018 [185] | Telehealth, mHealth | 3° | Comprehensive Continuous | ↑* | - |
| Reeves, 2016 [186] | HIT | 3° | Comprehensive Continuous Coordinated | - | ↑* |
| Regan, 2017 [187] | HIT, CDS | 3° | Continuous | - | ↑* |
| Richardson, 2019 [188] | Telehealth, CDS | 2°, 3° | Comprehensive | ↑* | - |
| Richter, 2015 [189] | Telehealth | 3° | Comprehensive Continuous Coordinated | - | ↑* |
| Ripley-Moffitt, 2015 [190] | HIT, CDS, other | 3° | Comprehensive | ↑* | - |
| Rollman, 2017 [191] | HIT, CDS | 3° | First Contact Continuous Coordinated | - | ↑* |
| Rose, 2019 [192] | HIT | 4° | Coordinated | - | ↑* |
| Satre, 2019 [193] | Telehealth, HIT, patient portals | 3° | Comprehensive Continuous | ↑* | - |
| Scheuner, 2014 [194] | CDS, other | 2° | Comprehensive | ↑* | - |
| Schiff, 2019 [195] | Telehealth, HIT | 2°, 3° | First Contact Comprehensive Coordinated | ↑* | - |
| Schlittenhardt, 2016 [196] | Telehealth | 3° | Comprehensive Continuous Coordinated | ↑* | ↑* |
| Senft, 2018 [197] | HIT | 3° | Coordinated | ↑* | - |
| Sequist, 2018 [198] | HIT, CDS | 3° | Comprehensive | ↑* | - |
| Shade, 2015 [199] | HIT, patient portals, CDS | 2°, 3° | Comprehensive Continuous Coordinated | ↑* | - |
| Shah, 2019 [200] | HIT, CDS | 1° | Comprehensive | ↑* | - |
| Shaibi, 2018 [201] | HIT | 2° | Comprehensive | ↑* | ↑* |
| Shaikh, 2014 [202] | Telehealth | 1°, 2°, 3° | First Contact Comprehensive Continuous | ↑* | - |
| Shane-McWhorter, 2014 [203] | Telehealth, HIT, CDS | 3° | Comprehensive Continuous | ↑* | - |
| Sharifi, 2014 [204] | HIT, CDS | 2°, 3° | Comprehensive Coordinated | - | - |
| Sharifi, 2017 [205] | HIT, CDS | 3° | Comprehensive Continuous | - | ↑* |
| Shuen, 2018 [206] | Telehealth, mHealth | 3°, 4° | First Contact Comprehensive Continuous | - | ↑* |
| Sidlow, 2015 [207] | HIT, CDS | 2° | Comprehensive | - | ↑* |
| Sigel, 2015 [208] | Telehealth, HIT | 2° | Comprehensive | - | ↑* |
| Siminerio, 2014 [209] | Telehealth | 3° | Comprehensive Continuous Coordinated | - | ↑* |
| Skolarus, 2017 [210] | mHealth, other | 3° | Continuous Coordinated | - | - |
| Smallwood, 2017 [211] | HIT, patient portals, CDS | 3° | Comprehensive | ↑* | - |
| Smania, 2016 [212] | mHealth | 2° | Comprehensive | - | ↑* |
| Smith, 2016 [214] | Telehealth | 3° | First Contact Comprehensive Continuous | - | ↑* |
| Smith, 2016 [213] | Telehealth | 3° | Comprehensive Continuous | - | ↑* |
| Sobota, 2015 [215] | HIT | 1° | Comprehensive | ↑* | - |
| Sood, 2018 [216] | Telehealth | 3° | Comprehensive Continuous Coordinated | ↑* | ↑* |
| Sperl-Hillen, 2018 [217] | Telehealth, HIT, CDS, other | 2°, 3° | Comprehensive | ↑* | ↑* |
| Stading, 2014 [218] | Telehealth | 3° | First Contact Comprehensive | ↑* | - |
| Steinberg, 2014 [219] | Telehealth, other | 3° | Comprehensive | ↑* | ↑* |
| Stevens, 2015 [220] | HIT, CDS | 4° | Comprehensive Coordinated | - | ↑* |
| Stockwell, 2017 [221] | mHealth | 2° | Comprehensive Continuous | - | ↑* |
| Stulberg, 2019 [222] | HIT | 2°, 3° | Comprehensive | ↑* | - |
| Sudhanthar, 2018 [223] | HIT, CDS | 1° | Comprehensive | - | - |
| Szilagyi, 2015 [224] | HIT, CDS | 1° | Comprehensive | ↑* | - |
| Talmi, 2014 [225] | HIT | 2° | Comprehensive | ↑* | - |
| Taveras, 2017 [226] | Telehealth, mHealth, HIT, CDS | 3° | First Contact Comprehensive Continuous | ↑* | - |
| Taylor, 2018[227] | Telehealth | 3°, 4° | Comprehensive Coordinated | - | - |
| Tetrault, 2020 [228] | Telehealth | 3° | First Contact Comprehensive | - | - |
| Tetuan, 2019 [229] | Telehealth, HIT | 1°, 3°, 4° | First Contact Coordinated | - | - |
| Thomas, 2018 [230] | HIT, CDS | 2° | Comprehensive | ↑* | - |
| Tieu, 2016 [231] | patient portals | 4° | Comprehensive Coordinated | ↑* | ↑* |
| Tsoh, 2018 [232] | mHealth | 3° | Comprehensive Continuous Coordinated | - | ↑* |
| Turner, 2018 [233] | HIT | 1°, 2°, 3°, 4° | Coordinated | ↑* | ↑* |
| Turvey, 2016 [234] | HIT, patient portals | 3° | Comprehensive Continuous | - | ↑* |
| Wagholikar, 2015 [235] | HIT, CDS | 3° | Comprehensive | ↑* | - |
| Walsh, 2017 [236] | Telehealth, HIT, CDS | 4° | Comprehensive | ↑* | - |
| Werk, 2019 [237] | Telehealth, HIT, CDS | 1° | Comprehensive | ↑* | - |
| Wise, 2017 [238] | CDS | 1° | Comprehensive Coordinated | ↑* | - |
| Wolin, 2015 [239] | Telehealth, other | 3° | Comprehensive Continuous | - | ↑* |
| Wolver, 2018 [240] | HIT | 4° | Comprehensive Continuous | - | ↑* |
| Woo, 2016 [241] | Telehealth, mHealth, HIT | 3° | Comprehensive Continuous | - | - |
| Wood, 2019 [242] | HIT, CDS | 2° | Comprehensive | ↑* | - |
| Wu, 2019 [243] | CDS | 2° | Comprehensive | - | ↑* |
| Yadav, 2019 [244] | HIT | 1°, 2° | Comprehensive | - | ↑* |
| Yakovchenko, 2019 [245] | Telehealth, mHealth | 3° | Comprehensive Continuous | ↑* | - |
| Yeboah-Korang, 2018 [246] | HIT, CDS | 2° | Comprehensive | ↑* | - |
| Yeung, 2016 [247] | Telehealth | 3° | Comprehensive Continuous | - | ↑* |
| Young, 2020 [248] | mHealth, HIT, WMD | 3° | Comprehensive | ↑* | - |
| Yu, 2018 [249] | mHealth, HIT | 3° | Comprehensive | ↑* | ↑* |
| Zera, 2015 [250] | HIT, CDS | 2° | Comprehensive | - | - |
| Zhao, 2019 [251] | mHealth, CDS | 2° | Comprehensive | - | ↑* |
| Zhong, 2018 [252] | HIT, patient portals | 3°, 4° | First Contact Coordinated | ↑* | - |
| Zieve, 2017 [253] | mHealth | 2°, 3° | Comprehensive Continuous | - | - |
| Zimet, 2018 [254] | HIT, CDS | 1° | Comprehensive | ↑* | - |
| Zimmerman, 2017 [255] | Telehealth, HIT, CDS | 1° | Comprehensive | ↑* | - |
| Zimmerman, 2017 [256] | Telehealth, HIT, CDS | 1° | Comprehensive | ↑* | - |
| Zimmerman, 2017 [257] | Telehealth, HIT, CDS | 1° | Comprehensive | ↑* | - |

^a^4C’s, first contact, comprehensive, continuous, and coordinated; CDS, clinical decision support; DHI, digital health intervention; HIT, Health information technology including electronic health records, electronic medical records, and health information exchange technologies; mHealth, mobile health; WMD, wireless medical devices

^b^Outcome summaries represent directionality of results and whether any results achieved statistical significance; ↑*, DHI improved outcome with *P*<.05 significance; -, not reported or improved outcome with *P*>.05 significance.
